# Harmonic voltage measurement error of the capacitor voltage transformer

**DOI:** 10.1371/journal.pone.0205231

**Published:** 2018-11-06

**Authors:** Jian Le, Hao Zhang, Chao Gao, Qian Zhou

**Affiliations:** School of Electrical Engineering, Wuhan University, Wuhan City, Hubei Province, China; Universita degli Studi della Tuscia, ITALY

## Abstract

This paper explores the mechanism for the harmonic voltage measurement error (HVME) of a capacitor voltage transformer (CVT) in a comprehensive way and develops a practical error correction method to improve the harmonic measurement performance of a CVT. The harmonic equivalent circuit (HEC) of CVTs with different types of dampers are established and the parameter value calculation methods of the circuit elements are presented. Characteristics of the individual inherent resonance mode of the HEC, including the resonant frequency, the resonant type and the circuit elements involved, are analyzed systematically. Centered on resonance mode formulation, an universal error correction method for CVTs in measuring harmonic voltage is proposed based on the piecewise fitting of the transformation ratio. A physical experimental platform available for low-voltage CVTs is developed in laboratory, which utilizes a 10-kV harmonic generator and a step-up transformer to provide harmonic signals with high voltage level. Moreover, a novel experimental scheme for testing the HVME of high-voltage CVTs is proposed based on alternating current/direct current (AC/DC) filters. Experiments are conducted on these experimental platforms to test the HVME of a 35-kV CVT in laboratory and a 525-kV CVT in a back-to-back DC converter station. The experimental results verify the validity of the built HECs of CVTs, the resonant mode analysis results and the resonant frequency calculation method. Finally, the effectiveness of the proposed harmonic error correction method is proved by several examples.

## 1. Introduction

CVT havs been widely adopted in high-voltage neutral grounding power networks to provide voltage signal with an apprciated accuracy to the energy metering system, relay protection system etc., due to its overwhelming advantages such as small volume, light weight, less maintenance and high margin of electric field intensity [1–3]. In China, CVT gradually occupies the majority of the market share of voltage measurement in from the begining of the 1980s, especially in the recent AC/DC ultrahigh-voltage project and the VSC-HVDC system [4, 5].

The hope that CVT can provide harmonic voltage signals with the same accuracy as that under the system fundamental frequency has been proven to be an illusion, via both the theoretical analysis and experimental verification; therefore, the relevant standard clearly states that “CVT cannot be used for harmonic voltage measurement”. Reference [6] discusses the problem of a CVT in measuring harmonics voltage, and notes that the measurement error mainly comes from the resonances in the circuit under particular harmonic frequencies. Based on the HEC of individual element, the influences of the burden rate and the damper structure on the frequency response of the transformation ratio of a CVT are analyzed quantitatively in [7], and the validity of the HEC formation and measurement error analysis are verified through simulation and field test of a 400kV CVT with a resonance-type damper. However, a CVT with a speedy saturation damper that is applied more widely in power system [8] has not been involved in this research. Reference [9] establishes the HEC of a CVT with a speedy saturation damper and, on this basis defines the HVME, while the mechanism for the error has not been studied. The influence laws of several factors that govern the harmonic measurement error of CVT are researched comprehensively in, e.g., [10–12]. References [10] and [11] focus on the effect of the stray and coupling capacitance on the harmonic voltage transfer function of the CVT, and [12] confirms that the measurement error mainly comes from the excitation impedance and the capacitance divider. Unfortunately, these studies are case specific and take only some of the influence factors into consideration, thus being unable to serve as the general basis for the quantitative analysis of the HVME. It shall be noted that method based on fractal theory [13, 14] is very beneficial for the formulation of eqivalent circuit of CVT.

Besides the theoretical analysis, several researches have focused on the experiment techonology for testing the HVME of CVTs. To learn the frequency response characteristics of the transformation ratio of a CVT, reference [15] proposes to apply harmonic voltage with a lower amplitude to the primary winding or the secondary winding of a CVT. Although a high-voltage harmonic source is no longer needed, the application of this scheme is very limited since that most CVTs do not allow to be energized from the secondary winding. In [16], an experimental arrangement that applies harmonic voltages to the midpoint of the capacitor voltage divider is presented, with the aim to derive the harmonic transformation ratio of a CVT. However, a harmonic source with high voltage level and large power capacity is still required.

It is well known that a resistor or a capacitor voltage divider is the most suitable candidate for the harmonics measuremnt in high-voltage systems. Unfortunately, these specialized devices are large in size and very expensive [17, 18] and are therefore inconvenient for field experiments. Some researchers have independently proposed to insert current transformers into the internal branches of a CVT [19, 20]. The harmonic voltage on the primary side can then be accurately measured according to outputs of the current transformers and the impedance-frequency characteristics of the circuit branches into which the current transformers are inserted. However, these methods are not applicable to CVTs that are already in operation.

This paper studies the harmonic voltage measurement error of a CVT in a comprehensive and in-depth way and, on this basis, presents a practical error correction method that can improve the harmonic measurement accuracy to an extent acceptable in engineering. The following innovative results are achieved: 1) In both the quantitative analysis and the influence law study of the measurement error, we concentrate on the resonant frequencies instead of analyzing the law of the influence factor individually. This change in disposal facilitates the revelation of the mechanism for measurement error greatly; 2) A physical experimental platform for testing the HVME of CVT is established. We propose a simple and effective scheme to address the problem of harmonic voltage amplification and attenuation, therefore improving the safety of the experimental platform and the accuracy of the experimental results; 3) We present a novel harmonic voltage measurement method based on the alternating current/direct current (AC/DC) filters for the high-voltage DC (HVDC) power transmission systems [21, 22]. This simple and practical method can provide accurate harmonic voltage reference signal for the determination of the HVME of CVT in high-voltage systems.

The remainder of this paper is structured as follows. In Section 2, the HECs of CVTs with different types of dampers are established. Section 3 presents the calculation of the resonant frequencies of the HECs. The harmonic voltage measurement performances of CVTs with different types of dampers are compared in Section 4. Section 5 presents the principle and application of the error correction method, simulation results verifying the effectiveness of this method are also provided in this section. Section 6 presents the scheme for the development of a physical experimental platform and the experiment results of the harmonic measurement error of a 35-kV CVT with a fast-saturation damper. Section 7 presents a method for measuring the harmonic voltage of HVDC power system and verifies the validity of the harmonic equivalent circuit of a 525-kV CVT. Section 8 applies the error correction method to correct the harmonic measurement error of a 35-kV CVT and a 525-kV CVT. Section 9 presents the conclusions.

## 2. Harmonic equivalent circuit of CVT

### 2.1 Harmonic equivalent circuit

**Fig 1:**
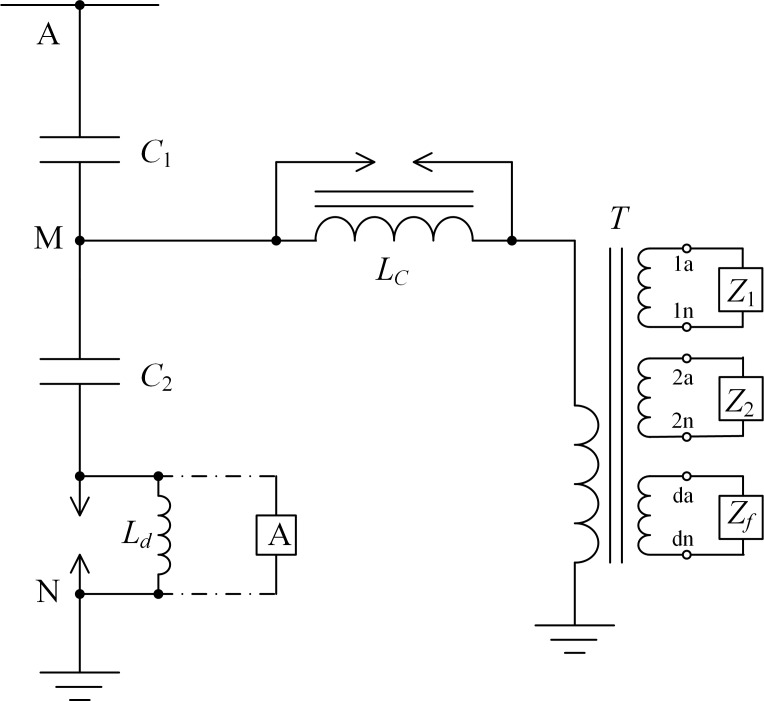
Structure of the CVT system.

As shown in Fig 1, the CVT system mainly consists of a capacitive voltage divider, an electromagnetic unit and the burden. The capacitive voltage divider is composed of the high-voltage capacitor *C*_1_ and the medium-voltage capacitor *C*_2_. The electromagnetic unit includes the compensation reactor *L*_*C*_, the intermediate transformer *T*, and the damper *Z*_*f*_. The damper can be connected to any one of the secondary windings.

**Fig 2:**
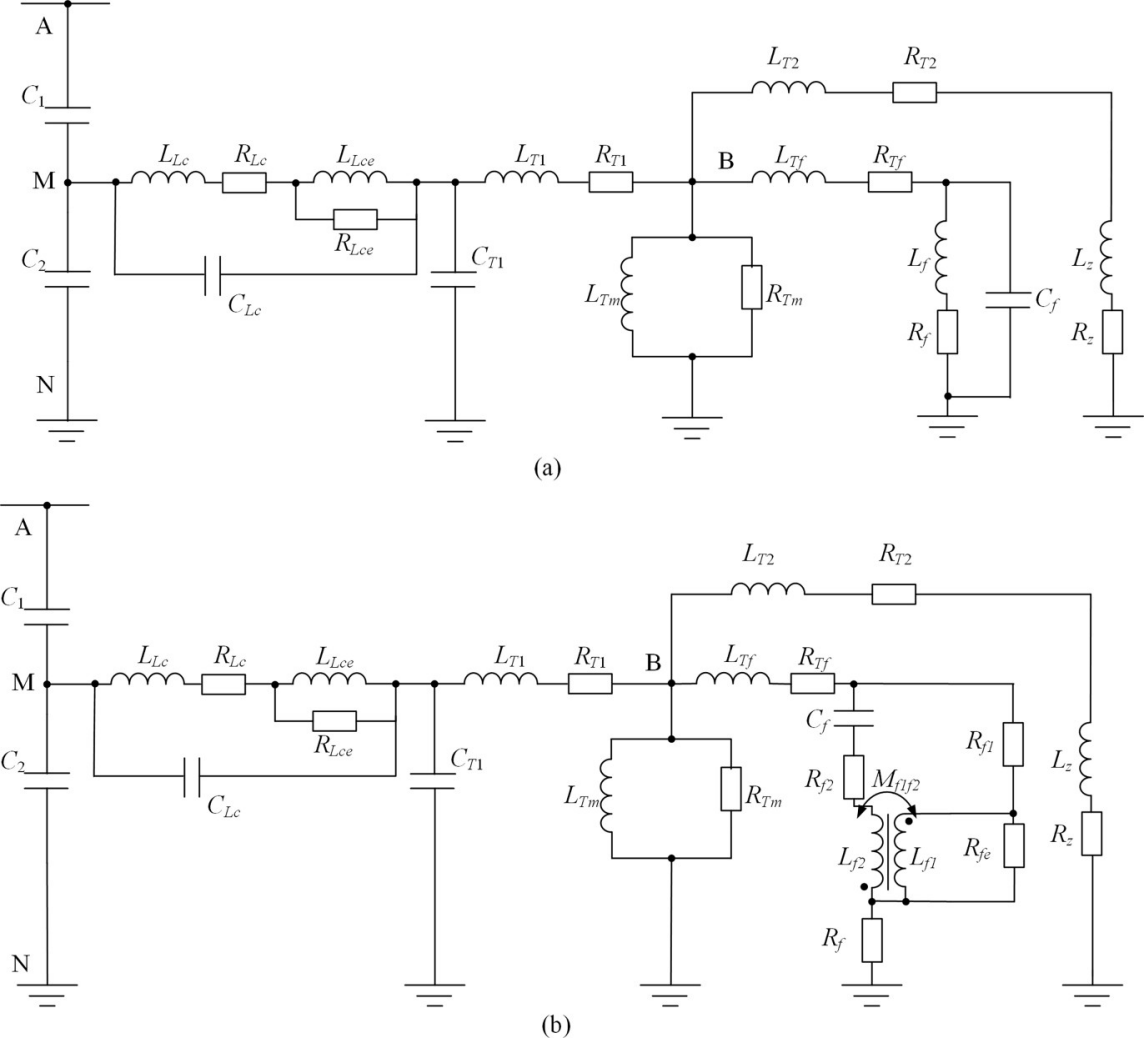
HECs of the CVTs. (a)With speedy saturation damper. (b) With resonance-type damper.

By ignoring the carrier extraction coil and converting the parameter values to the primary side, the harmonic equivalent circuits of the CVT with a resonant damper [7] and that with a speedy saturation damper have been built and are shown in Fig 2.

As seen in Fig 2, the equivalent circuit of the compensation reactor is composed of the inductance *L*_*Lc*_ and the resistance *R*_*L*_ of the winding, the inductance *L*_*Lce*_ and the resistance *R*_*Lce*_ of the iron core, and the stray capacitance *C*_*Lc*_. *L*_*T*1_/*R*_*T*1_, *L*_*T*2_/*R*_*T*2_, and *L*_*Tf*_ /*R*_*Tf*_ are the inductance/resistance of the primary winding, the measurement and protection winding, and the damper winding of the intermediate transformer, respectively. The measurement winding and protection winding are merged due to their same capacity and voltage level. *L*_*Tm*_ and *R*_*Tm*_ are the excitation inductance and resistance of the intermediate transformer, respectively. *C*_*T*1_ is the primary winding stray capacitance, and the stray capacitances of the secondary windings are ignored due to the relative small numbers of secondary winding turns, *L*_z_ and *R*_z_ are the burden inductance and resistance, respectively.

The major difference between the two equivalent circuits shown in Fig 2 is the HECs of the dampers with different structures. Specifically, in Fig 2(A) the HEC of the CVT with a speedy saturation damper, *L*_*f*_, *R*_*f*_, and *C*_*f*_ are the inductance, resistance and stray capacitance of the damper, respectively. As for the HEC of the CVT with a resonant damper, *R*_*f*1_, *R*_*f*2_ and *R*_*fe*_ are the resistances of the winding and iron of the damper transformer, respectively, *L*_*f*1_, *L*_*f*2_ and *M*_*f*1*f*2_ are the self-inductance and mutual inductance of the windings of the damper transformer, and *C*_*f*_ and *R*_*f*_ are respectively the capacitance and nonlinear resistance of the damper, as shown in Fig 2(B).

### 2.2 Calculation of the element parameter values

**Fig 3:**
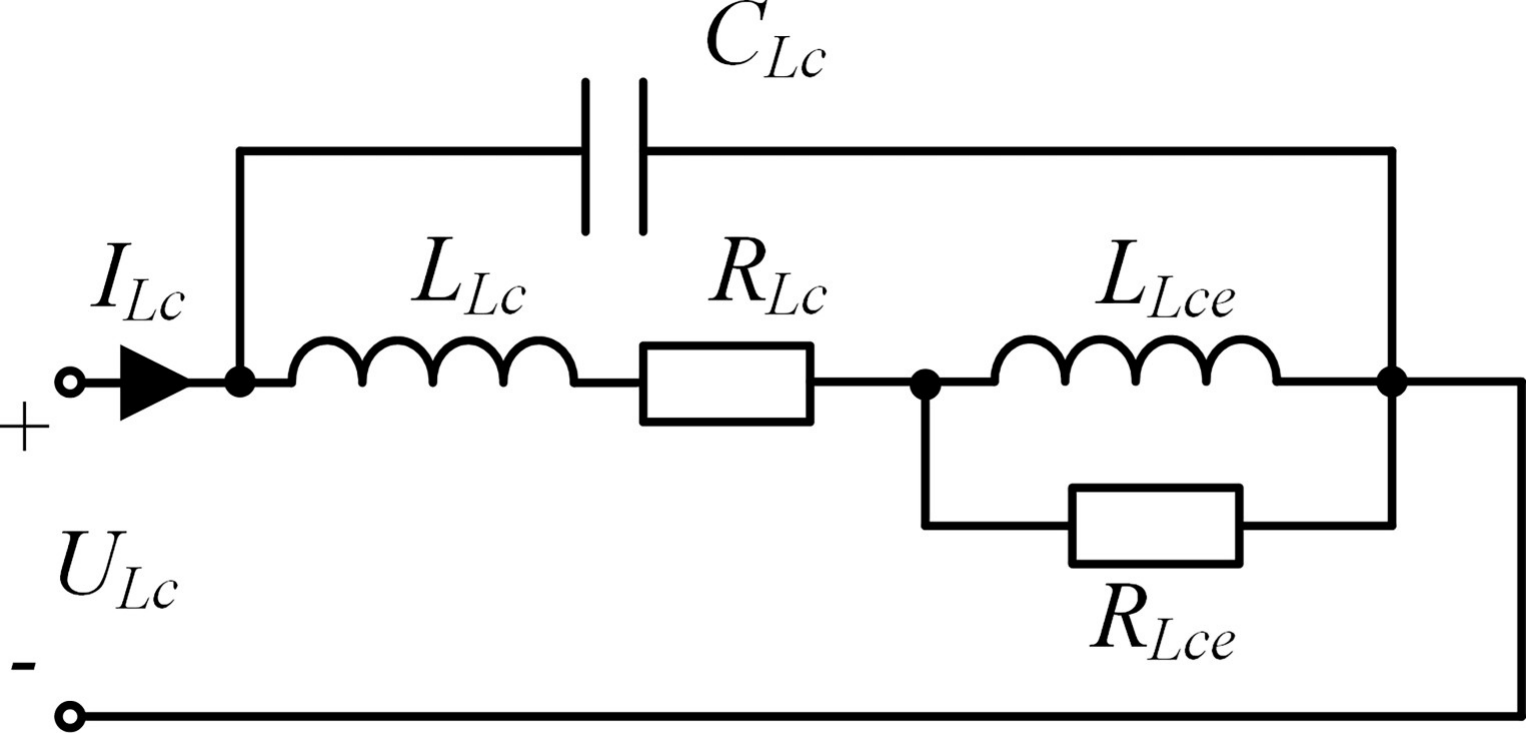
Element parameter calculation for the compensation reactor.

The parameter values of the elements in the HEC of the compensation reactor can be obtained via the corresponding experiments, based on the circuit shown in Fig 3.

To acquire the values of *R*_*Lc*_ and *R*_*Lce*_, we by turn inject a DC current and two AC currents with a frequency of 50 Hz and 100 Hz respectively, with the RMS values being the same as the rated current of the primary winding of the intermediate transformer, into the circuit and then record the voltage *U*_*Lc*_ under each test and the power loss under each of the AC current injection test.

After solving *R*_*Lc*_ accroding the measured DC voltage *U*_*Lc*_ and the injected DC current *I*_*Lc*_, *R*_*Lce*_ can then be obtained by solving:

$$R_{Lce}=P_{Lc(50)}/I_{Lc(50)}^{2}-R_{Lc}$$ (1)

where *P*_*Lc*(50)_ and *I*_*Lc*(50)_ are respectively the measured power loss and RMS value of the injected current in the 50 Hz AC current injection test.

The values of *L*_*Lc*_ and *L*_*Lce*_ can be calculated based on the recorded RMS values of the voltages and currents of the two AC current injection tests after the values of the resistances in Fig 3 have been derived. Next, we establish the simulation model of the compensation reactor using the Ansoft software according to its actual physical structure and dimensions; then, the value of the stray capacitance *C*_*Lc*_ can be estimated through proper selection of the material attribute and the setting of the boundary conditions.

As for the elements in the HEC of the intermediate transformer, the values of the leakage inductance *L*_*T*1_, *L*_*T*2_, and *L*_*Tf*_ and the leakage resistance *R*_*T*1_, *R*_*T*2_, and *R*_*Tf*_ of the corresponding winding, the excitation inductance *L*_*Tm*_ and the resistance *R*_*Tm*_ can all be known from the nameplate values provided by the manufacturer. The value of the stray capacitances *C*_*T*1_ can be acquired in the same way as that for the estimation of *C*_*Lc*_.

The burden inductance *L*_*z*_ and resistance *R*_*z*_ can be calculated by solving:

$$R_{z}=\frac{1}{2}\frac{U_{zN}^{2}}{\eta S_{zN}}\times \cos\varphi \times k_{z}^{2}$$ (2)

$$L_{z}=\frac{1}{2}\times \frac{1}{2\pi f_{1}}\frac{U_{zN}^{2}}{\eta S_{zN}}\times \sin\varphi \times k_{z}^{2}$$ (3)

where *U*_*zN*_ = $100/\sqrt{3}$V and *S*_*zN*_ = 100 VA are the rated voltage and rated capacity of the measurement and protection secondary winding, respectively. *η* is the burden rate and cos*φ* is the power factor of the burden, which has a normal value of 0.8 lagging. *f*_1_ = 50 Hz, and *k*_*z*_ is the open-circuit transformation ratio of the voltage between the primary winding and the two secondary windings of the intermediate transformer.

The value of the inductance *L*_*f*_ and the resistance *R*_*f*_ of the speedy saturation damper shown in Fig 2(A) can be easily calculated based on the actual values of these two elements provided by the manufacturer and the open-circuit transformation ratio of the voltage between the primary winding and the secondary winding to which the damper connectes. Similarly, the value of the stray capacitances *C*_*f*_ can be estimated using the Ansoft software. The situation is relatively complex for the resonant damper shown in Fig 2(B), the transformer winding resistances *R*_*f*1_and *R*_*f*2_can be obtained via the four-point measurement method, i.e., injecting a DC current with a value that is the rated current of the primary winding of the transformer into each of the windings. The values of *C*_*f*_ and *R*_*f*_ can be known from the nameplate, and the values of the iron core resistance *R*_*fe*_, the winding self-inductance*L*_*f*1_and*L*_*f*2_, and the winding mutual inductance *M*_*f*1*f*2_ can be determined by conducting regular short-circuit and open-circuit tests on the damper transformer.

### 2.3 Parameter value calculation results

Reference [7] provides all the parameter values of the HEC of a 400kV CVT with a resonant damper; we summarize the parameter values of all the elements besides this damper in Table 1 and specificlly list those of the filter in the left column of Table 2. Although we have obtained the parameter values of the elements of another 400kV CVT with a speedy saturation damper using the method introduced in the above subsection, for the purpose of performance comparison, the parameter values listed in Table 1 are used for the elements except for the speedy saturation damper, while those of this damper are separately given in the right column of Table 2.

**Table 1:** Parameter values of the common elements of the two CVTs.

Capacitor divider	HV capacitance *C*_1_	3.18nF	
	MV capacitance *C*_2_	54nF	
	Equivalent capacitance *C*_1_+*C*_2_	57.18nF	√
Compensation reactor	Winding resistance *R*_*Lc*_	766Ω	√
	Core resistance *R*_*Lce*_	9.288 MΩ	√
	Winding inductance *L*_*Lc*_	24.71H	√
	Core inductance *L*_*Lce*_	148.9H	√
	Stray capacitance *C*_*Lc*_	61.17pF	
Intermediate Transformer	Leakage resistance *R*_*T*1_	1403Ω	√
	Leakage inductance *L*_*T*1_	1.27H	√
	Leakage resistance *R*_*T*2_	1054Ω	√
	Leakage inductance *L*_*T*2_	6.95H	√
	Leakage resistance *R*_*Tf*_	5904Ω	√
	Leakage inductance *L*_*Tf*_	6.18H	√
	Excitation inductance*L*_*Tm*_	53kH	√
	Excitation resistance *R*_*Tm*_	1070Ω	√
	Stray capacitance *C*_*T*1_	267pF	
Rated burden	Resistance *R*_*z*_	645.2kΩ	√
	Inductance*L*_*z*_	1.54kH	√

**Table 2:** Parameter values of the two different dampers.

Resonant damper		Speedy saturation damper		
Damper resistance *R*_*f*_	37.5Ω	Stray capacitance *C*_*f*_	30pF	
Damper capacitance *C*_*f*_	15.4nF	Damper resistance *R*_*f*_	2725Ω	√
Resistance *R*_*f*1_	2731Ω	Damper inductance *L*_*f*_	3002H	√
Resistance *R*_*f*2_	240.6Ω			
Self-inductance *L*_*f*1_	529H			
Self-inductance *L*_*f*2_	5.2H			
Mutual inductance*M*_*f*1*f*2_	42.3H			
Core resistance *R*_*fe*_	13.8 MΩ			

## 3. Resonance mode analysis

Under the system fundamental frequency, a series resonance in the circuit, which is inherently formulated by the electromagnetic unit and the capacitive voltage divider of the CVT, can maintain a constant secondary-side output voltage under any burden condition, thus guaranteeing a satisfying voltage measurement accuracy. It is now well known that the large error in the measurement of harmonic voltage using a CVT originates from several resonances that are formulated by the stray capacitances and the corresponding inductive element in the circuit. However, the characteristics of the individual resonance mode, such as the resonant frequency and the circuit element involved, have not yet been researched comprehensively.

### 3.1 CVT with a speedy saturation damper

**Fig 4:**
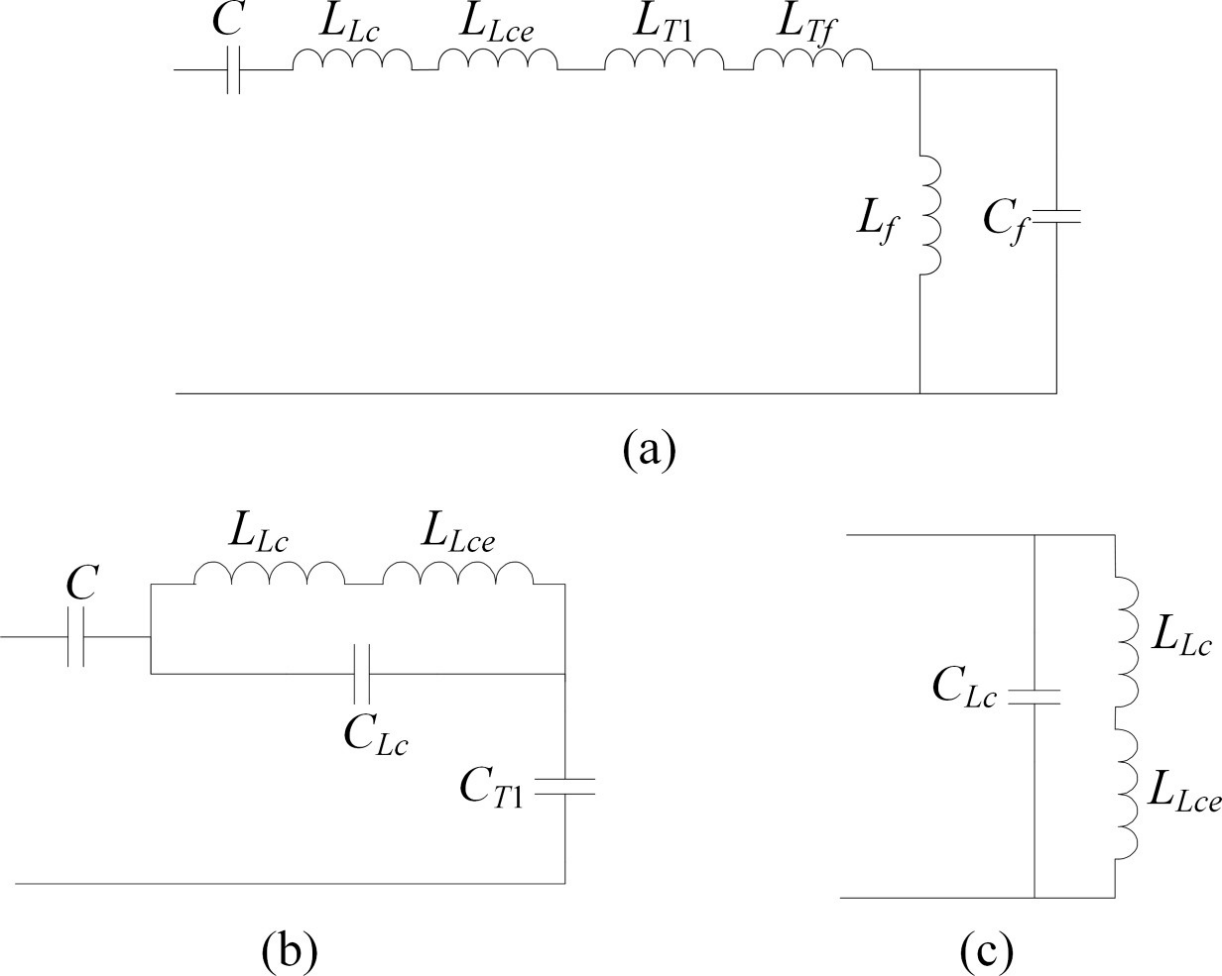
Resonant circuits of the CVT with a speedy saturation damper. (a) Resonant circuit 1. (b) Resonant circuit 2. (c) Resonant circuit 3.

After analyzing the HEC of the CVT with a speedy saturation damper shown in Fig 2(A), three resonance modes can be detected, and the relevant resonance circuits are presented in Fig 4.

From Fig 4(A), we can learn that the first resonance mode is of a series type and is caused by the Thevenin equivalent capacitance *C* of the capacitive voltage divider, winding and iron core inductance of the compensation reactor, winding leakage inductance of the intermediate transformer and the speedy saturation damper. The resonant frequency of resonant circuit 1 can be sovled using the parameter values listed in Table 1 and Table 2 as:

$$\begin{array}{l} X_{C}=\frac{1}{57.18\times 10^{-9}\times \omega }(\Omega )\\ X_{L}=\frac{3000\times \omega }{1-\omega^{2}\times 9\times 10^{-8}}+181.06(\Omega )\\ f_{1}=\omega /2\pi =11.8(\mathrm{Hz}) \end{array}$$

As seen from the resonance circuit shown in Fig 4(B), the second resonance mode is also of a series type that is determined by the equivalent capacitance *C*, winding and iron core inductance and the stray capacitance of the compensation reactor, and the stray capacitance in the primary winding of the intermediate transformer. The resonant frequency of this resonant mode is:

$$\begin{array}{l} X_{C}=\frac{1}{2.6576\times 10^{-10}\times \omega }(\Omega )\\ X_{L}=\frac{173.61\times \omega }{1-\omega^{2}\times 1.06197\times 10^{-8}}(\Omega )\\ f_{2}=\omega /2\pi =668(\mathrm{Hz}) \end{array}$$

We can see from Fig 4(C) that the third resonant mode is formulated by the paralleling inductance and stray capacitance branches of the compensation reactor. It is obviously a parallel-type resonance, with the resonant frequency being:

$$\begin{array}{l} X_{C}=\frac{1}{61.17\times 10^{-12}\times \omega }(\Omega )\\ X_{L}=173.61\times \omega (\Omega )\\ f_{2}=\omega /2\pi =1544.4(\mathrm{Hz}) \end{array}$$

### 3.2 CVT with a resonant-type damper

Similarly, five resonance modes of the HEC of the CVT with a resonant damper can be found in Fig 2(B), and Table 3 summarizes the resonant frequency, resonant circuit and the elements involved in each resonance mode.

**Table 3:** Resonance modes of the CVT with a resonant damper.

	Resonance mode 1	Resonance mode 2	Resonance mode 3	Resonance mode 4	Resonance mode 5
Resonant type	Series	Series	Paralleling	Paralleling	Series
Resonant frequency(Hz)	22.96	105.88	398.46	1544.4	3235.57
Elements involved	C, R, T & D*	C, R, T & D	T & D	R	C, R, T & D
Resonant circuit	see Fig 5(A)	see Fig 5(B)	see Fig 5(C)	see Fig 5(D)	see Fig 5(E)

*C = Capacitive voltage divider, R = Compensation reactor, T = Intermediate transformer, D = Damper.

**Fig 5:**
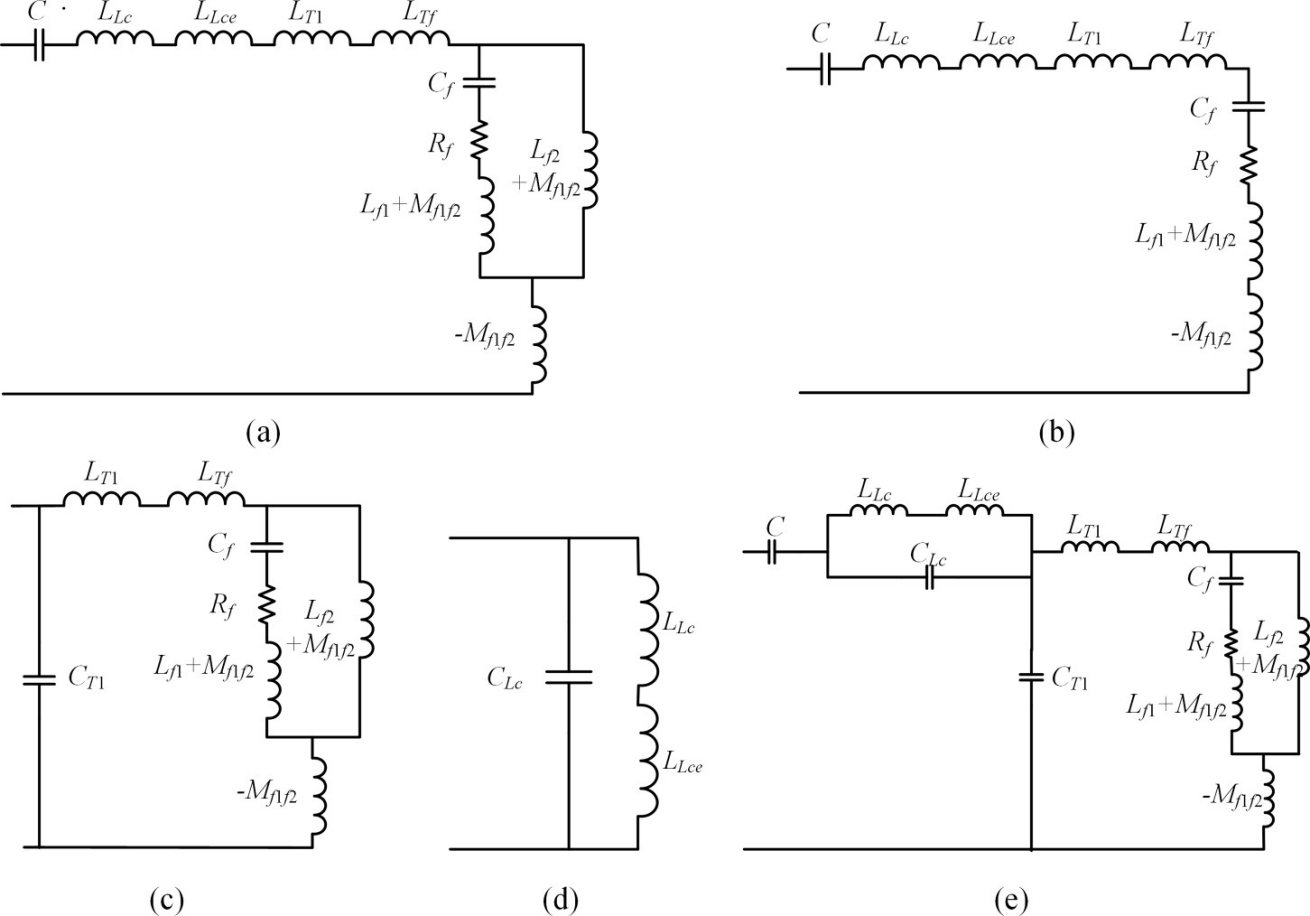
Resonant circuits of the CVT with a resonant-type damper. (a) Resonant circuit 1. (b) Resonant circuit 2. (c) Resonant circuit 3. (d) Resonant circuit 4. (e) Resonant circuit 5.

## 4. Frequency-response characteristics of the transformation ratio

**Fig 6:**
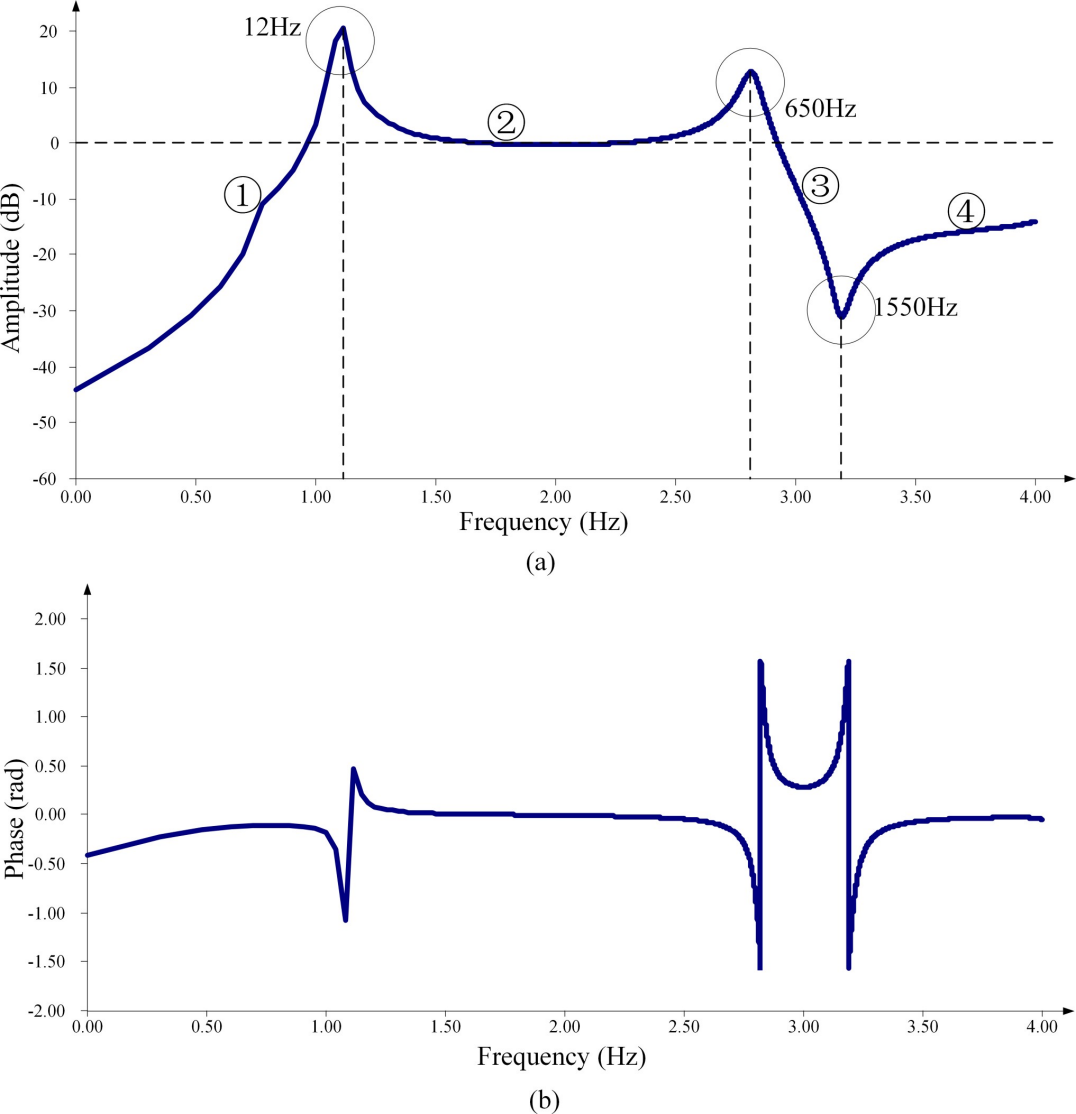
Frequency response characteristics of the ratio of the CVT with a speedy saturation damper. (a) Amplitude-frequency characteristic. (b) Phase-frequency characteristic.

**Fig 7:**
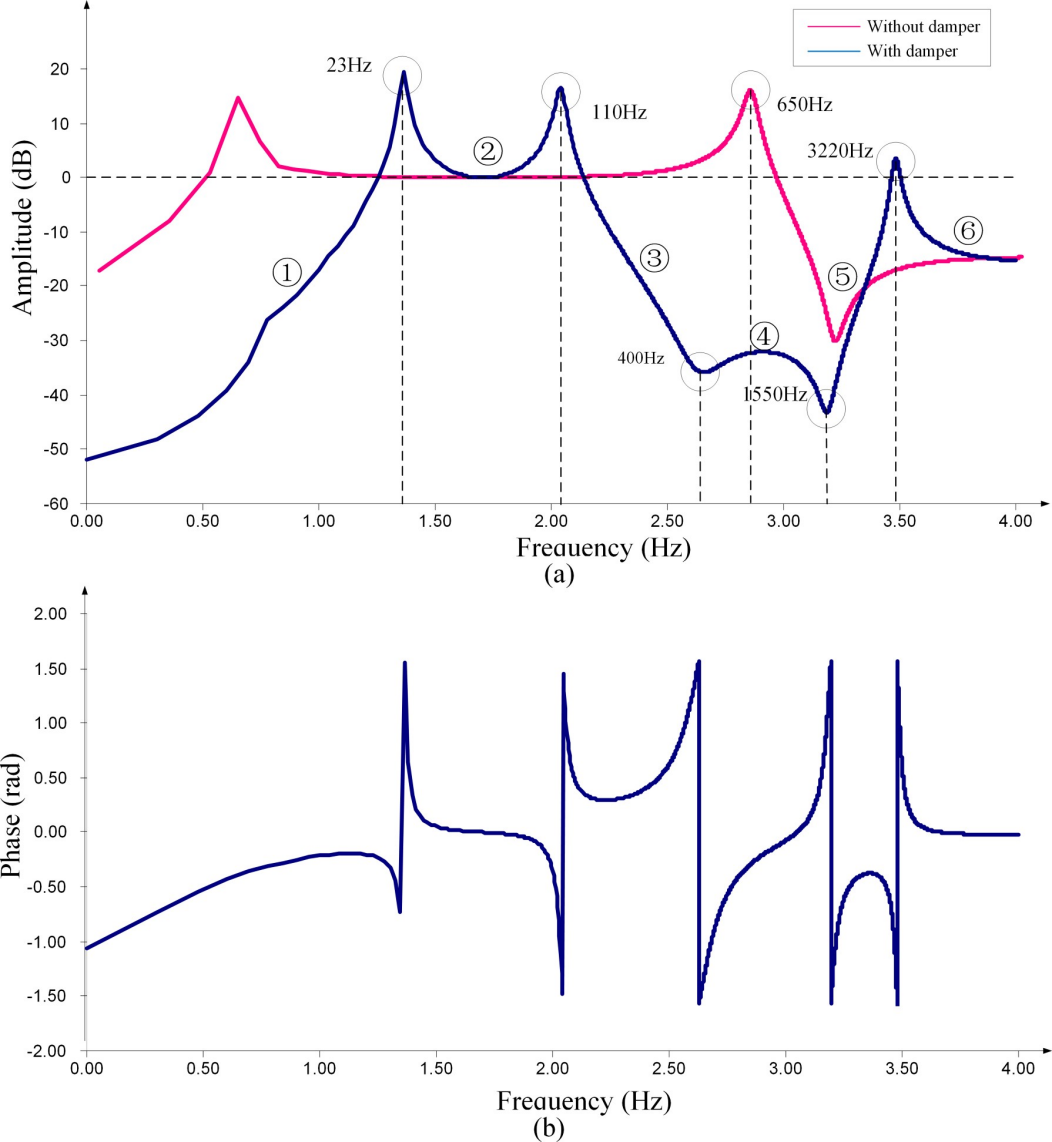
Frequency response characteristics of the ratio of the CVT with a resonant-type damper. (a)Amplitude-frequency characteristic. (b) Phase-frequency characteristic.

The simulation models of CVTs with different types of dampers have been built using the PSCAD software. Fig 6 and Fig 7 respectively illustrates the simulated frequency response characteristics of the voltage transformation ratio. In these figures, the x-axis is at the logarithmic scale, and the y-axis of the amplitude-frequency response figures is in units of dB. The amplitude of the transformation ratio is normalized according to the rated transformation ratio.

### 4.1 CVT with a speedy saturation damper

Fig 6(A) clearly shows that there are three extreme value points in the amplitude-frequency response characteristic of the transformation ratio for a speedy saturation damper. The two extreme value points locating at 12 Hz and 650 Hz respectively are of an amplitude larger than 0 dB, which indicates the formation of a series resonance in the HEC of the CVT. These results show excellent agreement with the analysis in Section 3.1, wherein the calculated resonance frequencies of the two series resonance modes is respectively 11.8 Hz and 668 Hz. The prediction of the existence of the parallel resonance mode is also well proven by the third extreme value point, with the amplitudes being smaller than 0 dB in Fig 6(A), and the simulated resonance frequency of 1550 Hz is very close to the calculated value of 1544.4 Hz.

### 4.2 CVT with a resonant-type damper

Similarly, we conduct a comparison of the simulated results (shown in Fig 7) with the theoretical analysis results (listed in Table 3) of the frequency response characteristic of the CVT with a resonant-type damper; the conclusions are summarized in Table 4.

**Table 4:** Comparison of the resonance modes of CVT with a resonant damper.

		Resonant type	Resonant frequency(Hz)
Resonance mode 1	Theoretical	Series	22.96
	Simulated	Amplitude>0 dB	23
Resonance mode 2	Theoretical	Series	105.88
	Simulated	Amplitude>0 dB	110
Resonance mode 3	Theoretical	Paralleling	398.46
	Simulated	Amplitude<0 dB	400
Resonance mode 4	Theoretical	Paralleling	1544.4
	Simulated	Amplitude<0 dB	1550
Resonance mode 5	Theoretical	Series	3235.57
	Simulated	Amplitude>0 dB	3220

Table 4 shows that the correctness of the theoretical analysis regarding the characteristics of the resonance modes of the HEC, including the resonant type and resonant frequency, is proven by the simulated frequency response characteristics

### 4.3 Harmonic voltage measurement error analysis

Based on the theoretical analysis and simulation results in the above sections, it can be concluded that if we use the rated voltage transformation ratio at the system fundamental frequency (50 Hz in this paper) to calculate the harmonic voltage at the primary side of the CVT, a large error in either the measured amplitude or the phase is inevitable due to the serious nonlinear behavior of and the large gap between the actual and rated values in the amplitude and phase of the transformation ratio frequency response, which is governed by the inherent resonance modes of the HEC of the CVT.

## 5. Harmonic measurement error correction method

At present, CVT is still the most widely used voltage measurement device in high-voltage systems, so it is of important practical value to study the practical HVME correction method to provide relatively accurate and reliable harmonic voltage measurement results. The key to improving the measurement accuracy is to accurately obtain the frequency response characteristic of the transformation ratio of CVT. This paper adopts the curve fitting method to fit the amplitude-frequency response characteristic of the transformation ratio of a CVT with a speedy saturation damper. Fig 6(A) shows that the amplitude-frequency characteristic at the resonance frequency has a rapid change and thus brings great difficulty to curve fitting. Therefore, we divide the fitting process into two steps. First, the resonant frequency is predicted, and then the frequency response characteristic is fitted piecewise, taking the resonance frequency as the boundary point.

### 5.1 Prediction of resonance frequency

The L-M-type BP neural network is adopted to predict the resonance frequency and the transformation ratio amplitude at the resonance frequency. Within the frequency range (less than 10000 Hz), the transformation ratio frequency response of the CVT with a speedy saturation damper has three resonance points; therefore, the output node number of the neural network number is 6, among which three nodes correspond to the three resonance frequencies and the remaining three nodes output the amplitudes of the transformation ratios at the resonance frequencies. The parameter values provided by the manufacturers, as shown in Table 1 and Table 2, are taken as the input variables. These variables are denoted with “√”, and 17 input variables are obtained in total.

Taking the typical values of the variables in Table 1 and Table 2 as the mean values and 2% of the mean values as the variance values, 100 groups of value samples are generated based on the normal distribution. Based on these values, the corresponding HEC is established in PSCAD to carry out the simulation calculation, and 100 groups of the amplitude-frequency response characteristics of the transformation ratio are obtained.

The training set is obtained by randomly selecting 50 groups of amplitude-frequency response characteristics, 25 groups of which are used to determine the suitable hidden layer number of the neural network, and the remaining 25 groups are taken as the validation sets. The commonly used leave-one-out cross method is used to determine the node number in the hidden layer, and the best node number of the hidden layer of the neural network is 12.

Table 5 presents the prediction results of the resonance frequency and the transformation ratio amplitude at the resonance frequency using the well-trained neural network. It can be seen that the designed neural network can fairly well predict the resonance frequency and the transformation ratio amplitude at the resonance frequency.

**Table 5:** Prediction of the resonant modes of the CVT with a speedy saturation damper.

Resonance mode	Resonance frequency			Ratio amplitude		
	Actual value (Hz)	Prediction value(Hz)	Error (%)	Actual value (dB)	Prediction value(dB)	Error (%)
1	12.975	13.095	0.925	8.482	8.576	1.11
2	689.984	689.985	0.0	14.477	15.217	5.11
3	1510.703	1567.455	3.76	-30.274	-31.352	3.56

### 5.2 The fitting of the amplitude-frequency response characteristic

After predicting the resonance frequency, the transformation ratio amplitude-frequency response characteristic is divided into several segments, taking the resonance frequency as the boundary point, and the corresponding neural networks are designed to fit these segments. The CVT with a speedy saturation damper has three resonance frequency points, so the amplitude-frequency response characteristic is divided into four segments, as shown in Fig 6(A). The L-M-type BP neural network is adopted to fit the amplitude-frequency response characteristic in these segments, the inputs and parameter samples in these segments are the same as the predicted resonance frequency in Part A of this section, and the node number of the hidden layer and the design of the output unit are different. For each segment of the amplitude-frequency characteristics, 0.004 decade (dec) is taken as the frequency interval to determine the corresponding output node number, and the leave-one-out cross method is used to determine the node number of the hidden layer in the neural network. The neural network design results are shown in Table 6.

**Table 6:** Design of the neural networks for the fitting of the frequency response characteristics.

Fitting object	Input node number	hidden layer node number	output node number
Segment ①	17	25	200
Segment ②	17	21	150
Segment ③	17	19	100
Segment ④	17	28	150

### 5.3 Analysis of the fitting results

**Fig 8:**
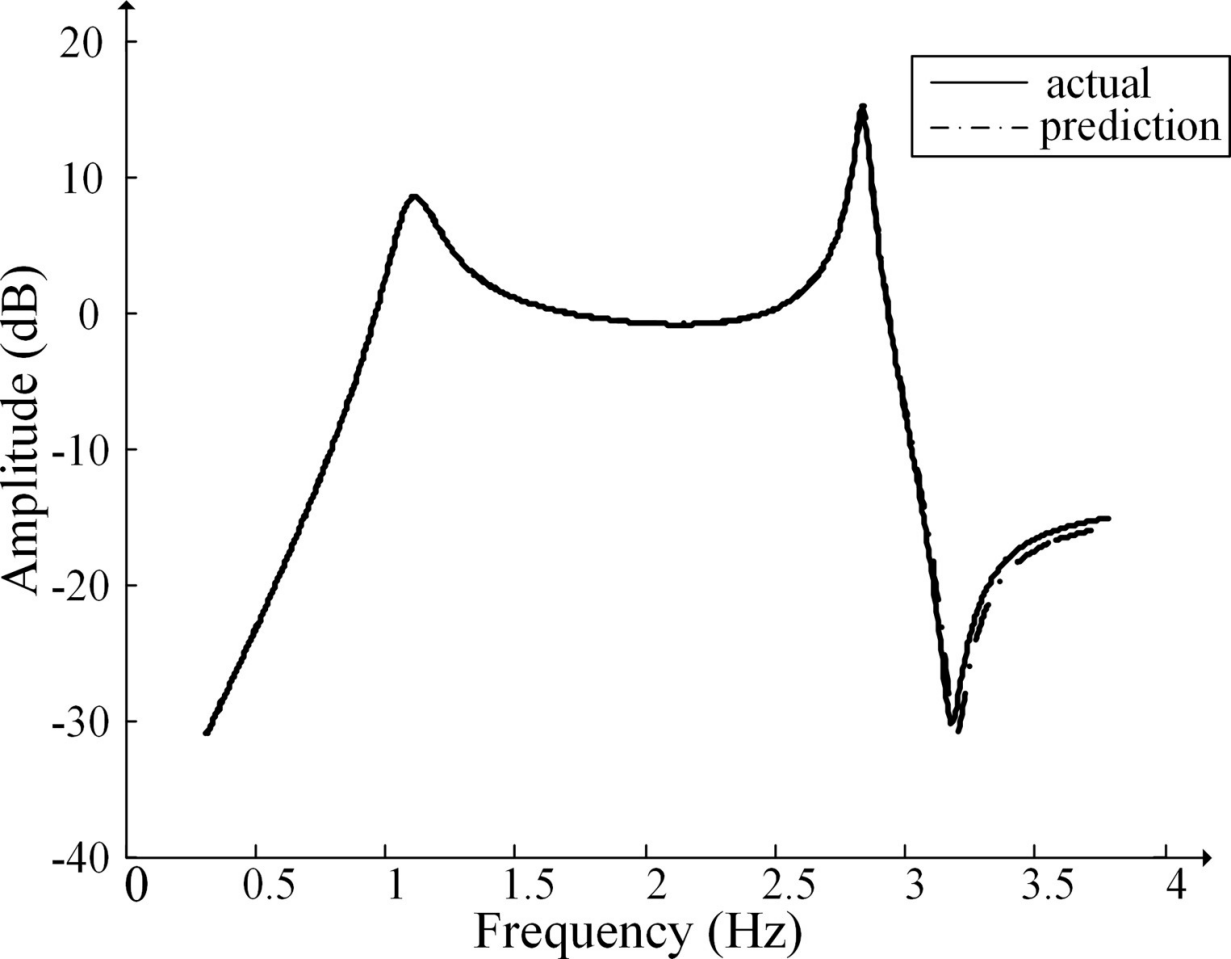
Fitting results of the amplitude of the transformation ratio.

A group of validation parameters are selected from the validation set, and Fig 7 presents the fitting results. Table 7 lists the comparison of the fitting results to the actual values within a part of the frequency. The decision coefficient for the amplitude-frequency characteristic curve fitting in Fig 8 is 0.99828. The prediction and fitting method can fairly well realize the prediction of the transformation ratio amplitude-frequency characteristic of the CVT and thus improve the accuracy of CVT harmonic voltage amplitude measurement.

**Table 7:** Fitting results of the amplitude-frequency response of CVT with a speedy saturation damper.

Frequency (Hz)	Actual value (dB)	Fitting results (dB)	Error (%)
100	-0.02071	-0.03482	68.09%
150	-0.79653	-0.79714	0.08%
200	-0.88137	-0.86266	-2.12%
250	-0.72488	-0.69204	-4.53%
300	-0.41592	-0.37101	-10.80%
350	0.031082	0.088049	183.28%
400	0.629597	0.699825	11.15%
450	1.413035	1.498716	6.06%
500	2.439797	2.544383	4.29%
550	3.807935	3.93727	3.40%
600	5.693182	5.859398	2.92%
650	8.413642	8.651041	2.82%
700	12.34333	12.79019	3.62%
750	14.10634	14.82924	5.12%
800	9.086895	9.520996	4.78%
850	4.341188	4.698706	8.24%
900	0.683054	1.047568	53.37%
950	-2.3229	-1.92282	-17.22%
1000	-4.93639	-4.48395	-9.17%
1050	-7.31057	-6.79119	-7.10%
1100	-9.5415	-8.93935	-6.31%
1150	-11.6988	-10.9949	-6.02%
1200	-13.8383	-13.009	-5.99%
1250	-16.011	-15.0259	-6.15%
1300	-18.27	-17.0895	-6.46%
1350	-20.6677	-19.2445	-6.89%
1400	-23.2506	-21.5407	-7.35%

## 6. Laboratory experiment results

### 6.1 Experimental platform

**Fig 9:**
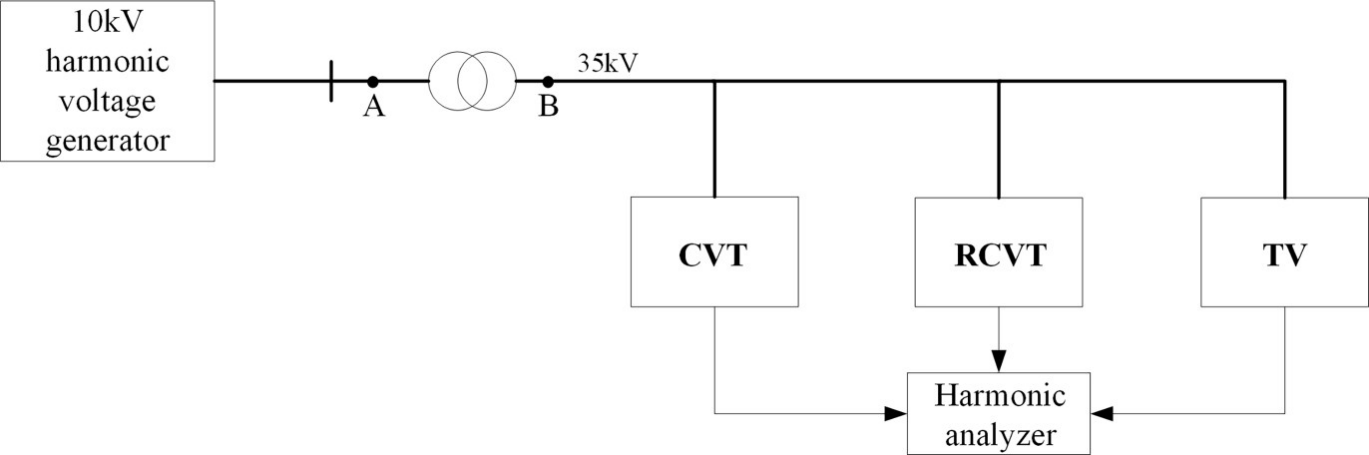
Structure of the experimental platform with a step-up transformer.

Fig 9 shows the circuit diagram of the experimental platform. During the experiment, the harmonic voltage is provided by a self developed high-capacity three-phase harmonic source with a rated line voltage of 10-kV. Considering the minimum voltage level of a CVT is a line voltage of 35-kV, a small capacity step-up transformer is employed to increase the harmonic voltage to 35-kV.

**Fig 10:**
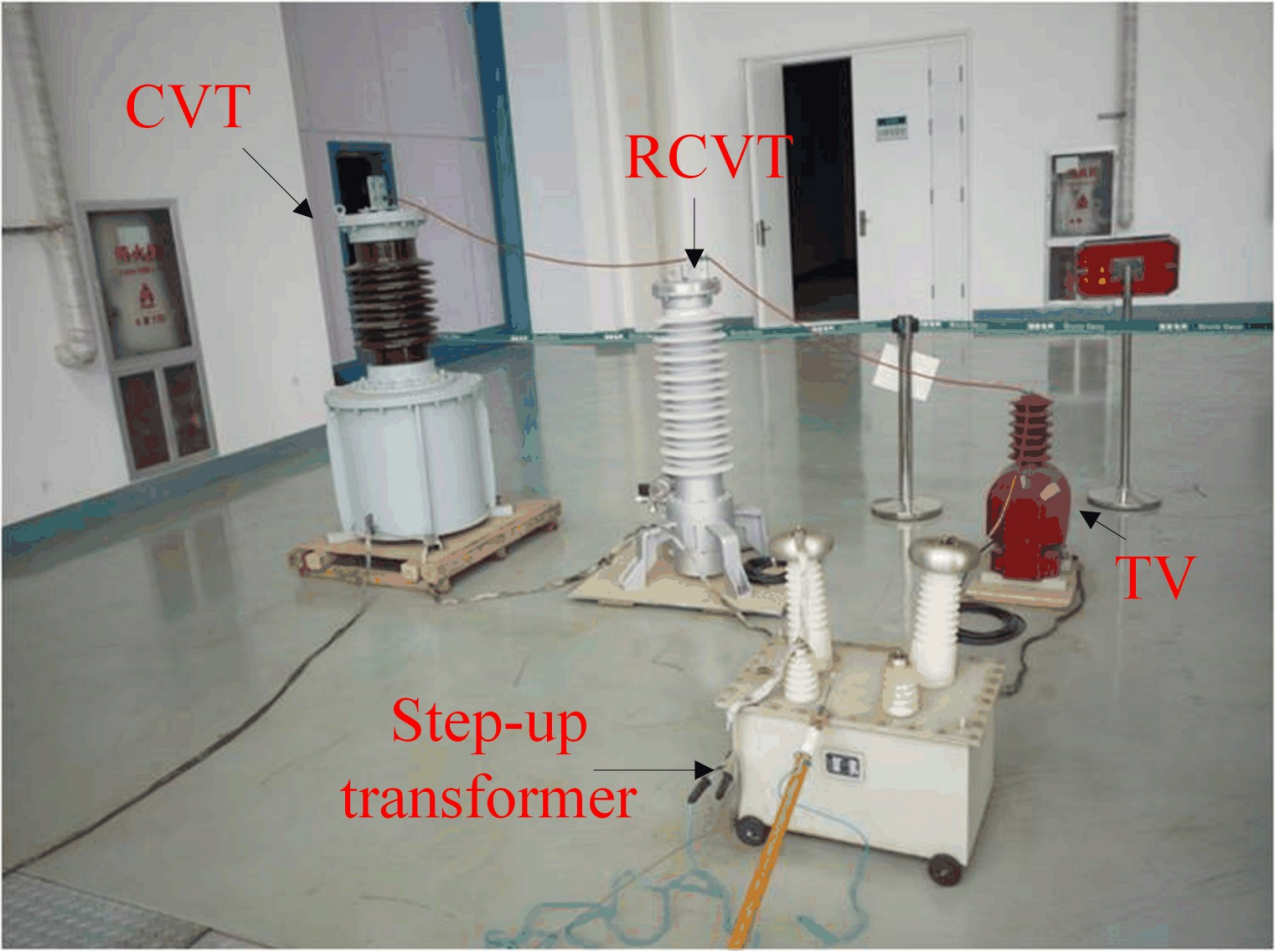
Arrangement of part of the experimental setup.

As shown in Fig 10 the arrangement of platform, a CVT with a speedy saturation damper, an electromagnetic voltage transformer (TV) and a resistive CVT (RCVT) are connected in parallel to one phase of the step-up transformer on the 35-kV side. The experiment is conducted to simultaneously analysis the harmonic measurement error of the CVT and that of the TV. The RCVT provides the actual harmonic voltage measurement on the 35-kV side to determine the harmonic measurement error. The outputs of these three devices are simultaneously connected to a Fluke 1760 power quality analyzer.

**Fig 11:**
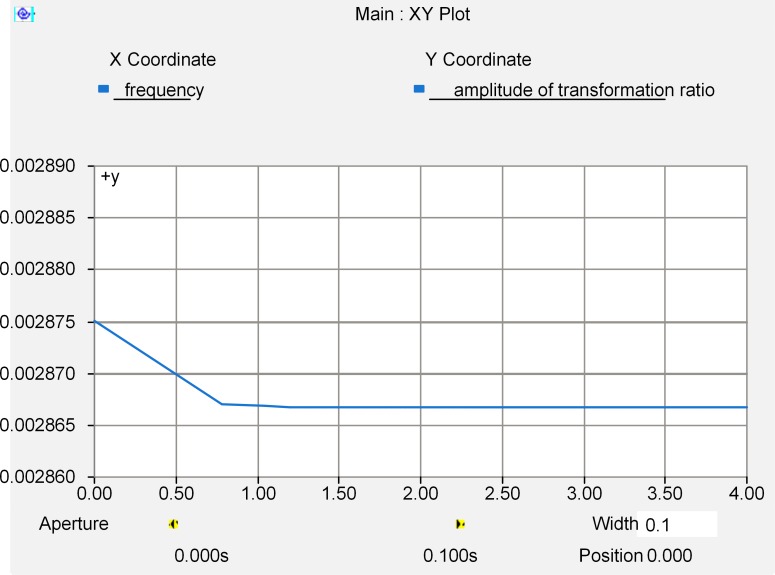
Amplitude-frequency response of the RCVT transformation ratio.

Fig 11 shows the amplitude-frequency response characteristics of the transformation ratio of the RCVT obtained by simulations, where the horizontal axis corresponds to the logarithmic scale frequency. We can see that the RCVT has a very wide frequency band with a rated fundamental transformation ratio of 100V/35,000V = 0.002857, thus can be used as the standard measurment device in the experiment.

### 6.2 Analysis of the experimental results and improvement plan

The 10-kV harmonic generator is controlled to output the rated fundamental voltage superimposed with the 2^nd^-31^st^ harmonic voltages with amplitudes of 1 kV (line voltage). The load of the RCVT is the input resistance of the power quality analyzer (1-MΩ), and the loads of the CVT and VT are the same. Experiments are conducted under four sets of loading conditions: (1) power factor: 1; load: 100% of the rated load (denoted by 100%+1); (2) power factor: 0.8 lagging; load: 100% of the rated load (denoted by 100%+0.8); (3) power factor: 1; load: 50% of the rated load (denoted by 50%+1); (4) power factor: 0.8 lagging; load: 50% of the rated load (denoted by 50%+0.8). Table 8 shows the 2^nd^-15^th^ harmonic voltage measurements on the secondary side of the RCVT, CVT and VT (due to the limited space, only the results for three of the four sets of loading conditions are presented).

**Table 8:** RMS values of the measured harmonic voltages.

Harmonic order	RCVT /V			CVT /V			VT/V		
	100%+1	100%+0.8	50%+0.8	100%+1	100%+0.8	50%+0.8	100%+1	100%+0.8	50%+0.8
2	7.7467	7.7679	7.8087	7.8050	7.8184	7.8758	7.6903	7.7729	7.8069
3	12.132	12.128	12.167	12.368	12.315	12.409	12.0795	12.2107	12.1218
4	22.796	22.527	23.073	23.832	23.439	24.191	22.6907	23.3205	23.0523
5	8.8914	8.8871	8.8851	9.6356	9.5443	9.6651	8.8616	8.9121	8.8710
6	4.4491	4.4531	4.4423	5.0469	4.9729	5.0710	4.4287	4.4387	4.4340
7	2.7864	2.7899	2.7779	3.4175	3.3440	3.4512	2.7771	2.7821	2.7729
8	1.9165	1.9190	1.9093	2.5683	2.4449	2.6333	1.9053	1.9097	1.9038
9	1.4444	1.4498	1.4434	2.2121	2.0052	2.3606	1.4402	1.4398	1.4361
10	1.1148	1.1169	1.1104	1.6170	1.3852	2.0170	1.1100	1.1103	1.1073
11	0.8943	0.8959	0.8948	0.7072	0.5472	0.7491	0.8902	0.8931	0.8847
12	0.7385	0.7400	0.7378	0.2020	0.1885	0.2339	0.7346	0.7370	0.7352
13	0.6160	0.6171	0.6163	0.1261	0.1233	0.1409	0.6131	0.6148	0.6044
14	0.5233	0.5241	0.5234	0.1637	0.1671	0.1675	0.5203	0.5219	0.5220
15	0.4570	0.4576	0.4571	0.1749	0.1984	0.2004	0.4542	0.4556	0.4440

In theory, the amplitude of the harmonic voltage on the secondary side of the RCVT is 5.7737 V when the amplitude of the harmonic voltage on it primary side is 3.5 kV/√3 and the transformation ratio is 0.002857, i.e., 3.5 kV×0.002857/√3 = 5.7737 V. However, Table 8 shows that under each loading conditions, the amplitude of the harmonic voltage on the secondary side of the RCVT differs significantly from the theoretical value and the amplitude of the harmonic voltages of some orders (e.g., 2^nd^-5^th^) are considerably amplified. For example, the amplitude of the 4^th^ harmonic voltage under the 100%+1 loading condition is 22.796 V. This result indicates that the 4^th^ harmonic voltage on the 35-kV side could be as high as 7.979 kV; this amplified harmonic voltage may threaten the safety of the experimental system. In contrast, harmonic voltages of some higher orders (6^th^-15^th^) are significantly attenuated. For example, the amplitude of the 15^th^ harmonic voltage under the 100%+1 loading condition is 0.4570 V, indicating that the amplitude of the 15^th^ harmonic voltage on the 35-kV side is only 160 V. The harmonic voltage with a too low amplitude may decrease the accuracy of the experiments to a certain extent. The phenomenon of harmonic voltages amplification and attenuation suggests that the orginal experimental platform scheme should be improved.

Table 9 lists the specific parameter values of the single-phase 10-kV/35-kV step-up transformer adopted in the original experiment platfrom. We can see that this transformer has a relatively small capacity and the short-circuit impedances under higher frequencies are very large. We decide to replace this transformer with a single-phase 10-kV/35-kV oil-immersed transformer, of which the specific parameter values are listed in Table 9.

**Table 9:** Parameters of the two step-up transformers.

Step-up transformer	Capacity (kVA)	Short-circuit impedance (p.u.)	Short-circuit loss (p.u.)	No-load loss (p.u.)
original	10	0.12	0.015	0.002
ordinary	1000	0.065	0.012	0.0014

**Fig 12:**
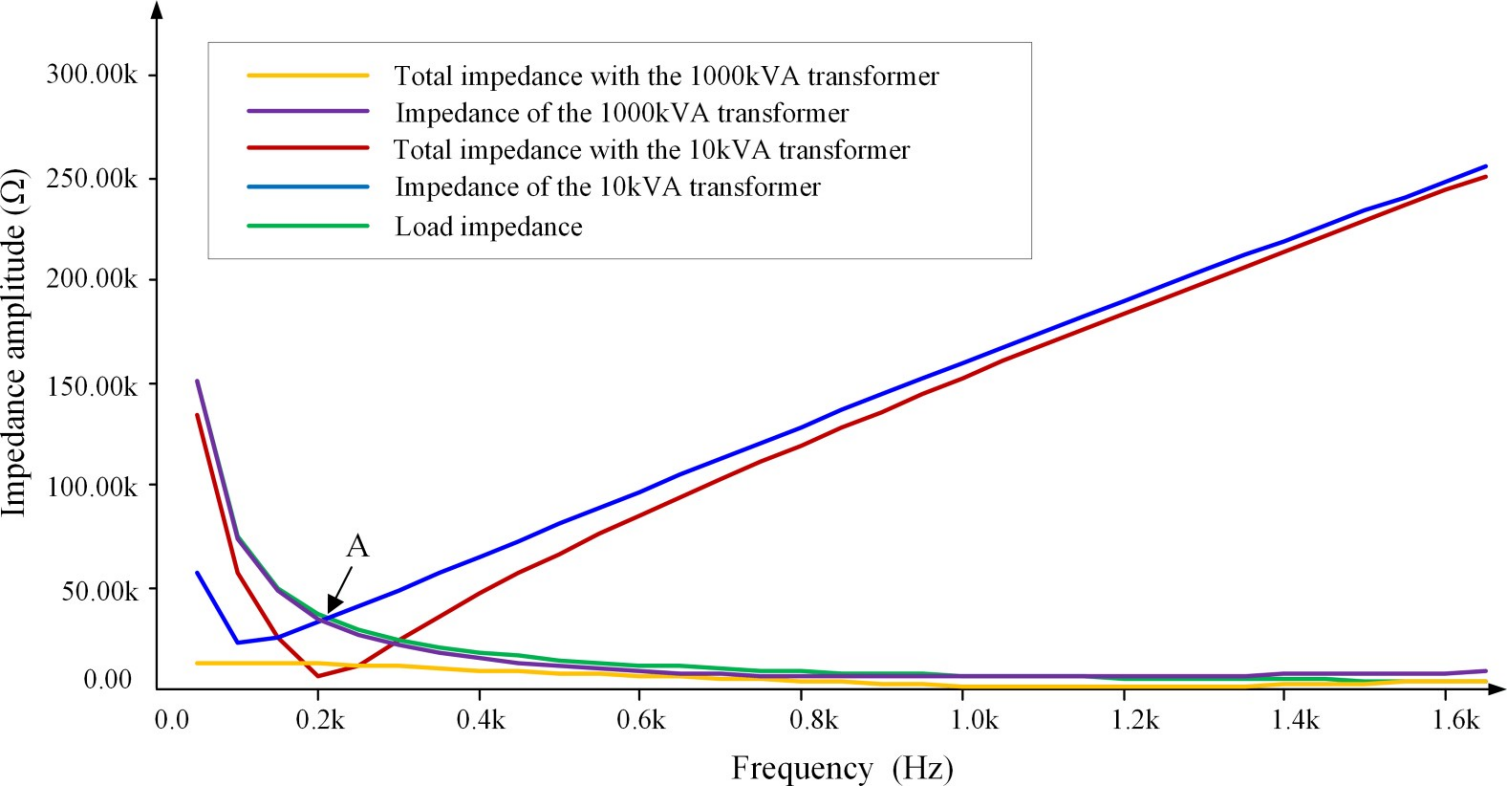
Impendence-frequency characteristics of the experimental platform circuit.

Fig 12 shows the inherent impedance of two step-up transformers with different capacities as well as the total impedance of the experimental platform circuit (observed from point A in Fig 9) and the amplitude-frequency response characteristics of the total load of the experimental platform (observed from point B in Fig 9) for these two transformers.

Fig 12 demonstrates that for the small-capacity step-up transformer (original one), both the total impedance and the transformer impedance increase as the frequency increases above 200 Hz, whereas the load impedance decreases to be far smaller than the transformer impedance. Thus the harmonic voltage mostly falls on the leakage reactance of the step-up transformer, resulting in significant attenuation of the harmonic voltage on the 35-kV side. In addition, the amplitude of the transformer impedance (inductive) is very close to that of the load impedance (capacitive) at point A in Fig 12 (where the frequency is approximately 200 Hz). Therefore, the experimental platform circuit will resonate, significantly amplifying the lower-order (2^nd^-5^th^) harmonic voltages near 200 Hz on the 35-kV side. This phenomenon is consistent with the experimental results shown in Table 8, which also demonstrates the safety risk and poor measurement accuracy of the experimental platform equipped with the original step-up transformer. In comparison, when applying a step-up transformer with a capacity of 1000 kVA, the transformer impedance is always smaller than the load impedance within the entire frequency range investigated. The simulation results show that for this transformer, no harmonic voltage amplification or attenuation occurs on the 35-kV side. This finding indicates that the experimental platform with a large-capacity step-up transformer is feasible.

### 6.3 Harmonic measurement error of the35-kV CVT

**Fig 13:**
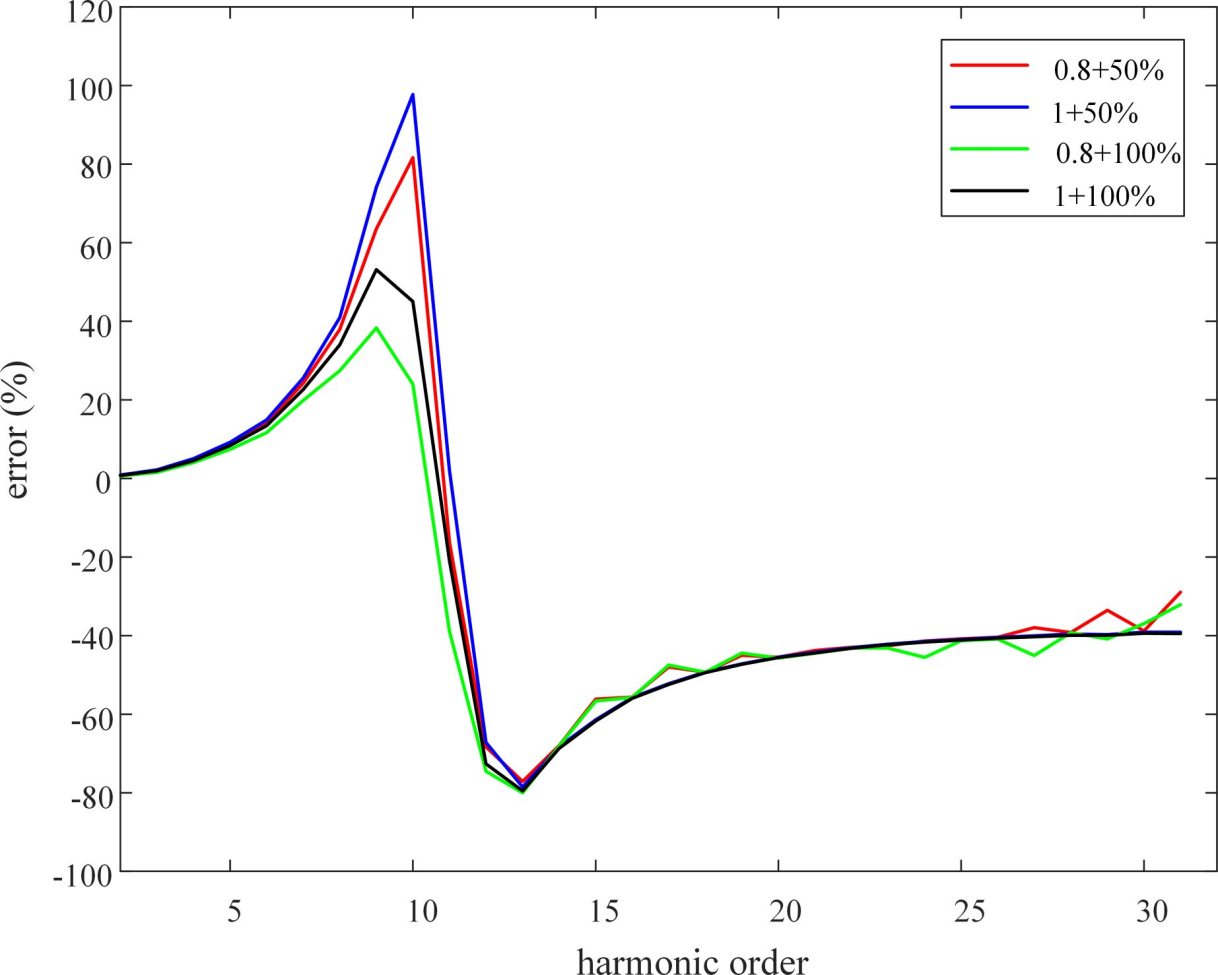
Harmonic voltage measurement error of the 35-kV CVT.

Table 10 lists the experimental results with respect to the secondary side of the CVT obtained under the 100%+1 loading condition. Fig 13 shows the harmonic measurement error under various sets of loading conditions. The harmonic measurement error is defined as:

$$error=\frac{U_{RCVT}(h)-U_{CVT}(h)}{U_{RCVT}(h)}\times 100\%$$

where *U*_*RCVT*_(*h*) and *U*_*CVT*_(*h*) represent the amplitude of the *h*^th^ harmonic voltage measured by the RCVT and CVT, respectively.

**Table 10:** Harmonic measurement error of the 35-kV CVT.

Harmonic voltage orders	Measurement error (%)	Harmonic voltage orders	Measurement error (%)	Harmonic voltage orders	Measurement error (%)
2	0.8598	3	1.9856	4	4.8436
5	8.7777	6	14.1526	7	24.2350
8	37.9226	9	63.5481	10	81.6521
11	-16.2789	12	-68.3040	13	-77.1423
14	-68.0002	15	-56.1596	16	-55.6452
17	-47.9928	18	-49.3004	19	-45.0349
20	-45.4857	21	-43.7971	22	-42.9837
23	-42.5665	24	-41.3738	25	-40.8241
26	-40.3929	27	-37.9508	28	-39.2155
29	-33.5749	30	-38.7925	31	-28.932

**Fig 14:**
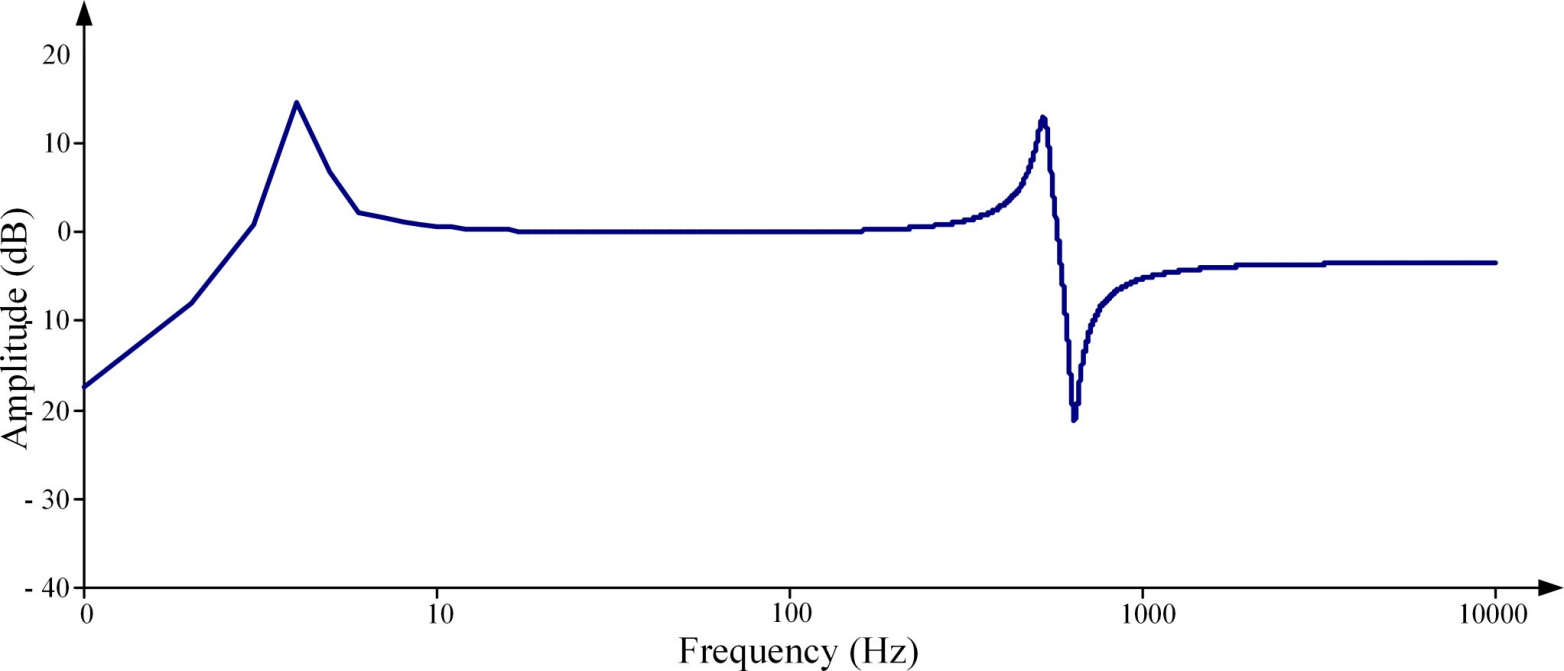
Amplitude-frequency characteristic of the transformation ratio of the 35-kV CVT.

Using the method presented in Section 2 of this paper, a harmonic equivalent circuit is established for this 35-kV CVT, and the parameters of the circuit components are obtained. The three resonant frequencies of the harmonic equivalent circuit are calculated as follows: *f*_1_ = 2.88 Hz, *f*_2_ = 525.55 Hz and *f*_3_ = 646.96 Hz. Fig 14 shows the amplitude-frequency response characteristics of the CVT transformation ratio obtained using PSCAD simulation.

The curve shown in Fig 14 has three extreme points, which correspond to frequencies of approximately 3 Hz, 500 Hz and 650 Hz. In addition, Fig 12 shows that the harmonic measurement error obtained from the experiment has two extreme points, which occur near the frequencies of the 10^th^ (500 Hz) and 13^th^ (650 Hz) harmonic components. No experiment is conducted on frequencies lower than 50 Hz. The extreme values of the harmonic measurement error indicate that the amplitude of the CVT transformation ratio also has extreme values. Thus, the theoretical analysis, simulation results, and experimental results with respect to the resonant mode and resonant frequency of the CVT with a speedy saturation damper are in very good agreement. Furthermore, Table 10 demonstrates a relatively large error in the harmonic voltage measurements using the CVT; specifically, the measurement error of the amplitude of the 10^th^ harmonic voltage reaches 81.65%, which once again confirms the statement that CVTs cannot be used to measure harmonics.

## 7. Field experiment on measuring the harmonic measurement error of a CVT

It is hard to perfrom the experiment on CVTs with voltage ratings of 110kV or above in laboratory, due to the technical difficulty and high investment in the development of a harmonic voltage generator with a high voltage level and a large power capacity. A convenient and practical method, which plays the same role as that of the RCVT, is the foundation to successfully conduct the field experiments on the harmonic voltage measurement error of CVTs with high voltage ratings.

### 7.1 Harmonic measurement method for high voltage systems

We propose a harmonic voltage measurement method that is used exclusively for HVDC and UHVDC power transmission systems with paralleling AC/DC filters. In this method, the current *i*_*zH*_ of the filter is measured and then decomposed using e.g. FFT technology. Then, based on the frequency characteristics of the filter impedance provided by the manufacturer, the amplitude and phase of the individual harmonic voltage can be calculated by:

$$U_{S(h)}∠\varphi_{S}{}_{\left(h\right)}=I_{ZH(h)}\cdot Z_{\left(h\right)}∠(\varphi_{\left(h\right)}+\theta_{ZH(h)})$$

where *I*_*ZH*(*h*)_ and *θ*_*ZH*(*h*)_ is the RMS value and phase angle of the *h*^*th*^ harmonic component of the current in the filter, respectively; *Z*_(*h*)_ and *φ*_(*h*)_ is respectively the amplitude and phase angle of the *h*^*th*^ harmonic impedance of the filter; and *U*_*S*(*h*)_ and *φ*_*S*(*h*)_ is respectively the RMS value and phase angle of the *h*^*th*^ harmonic component of the voltage of the bus to which the filter connects.

We can see from the above explanation that the proposed harmonic voltage measurement method is of simple calculation and easy implementation since that it requires the real-time current and impedance frequency characteristic of the AC/DC filter only. It should be noted that a current transformer generally has an appreciated frequency response characteristic to provide accurate harmonic current measurement signals [23].

### 7.2 Application of the harmonic measurement method

**Fig 15:**
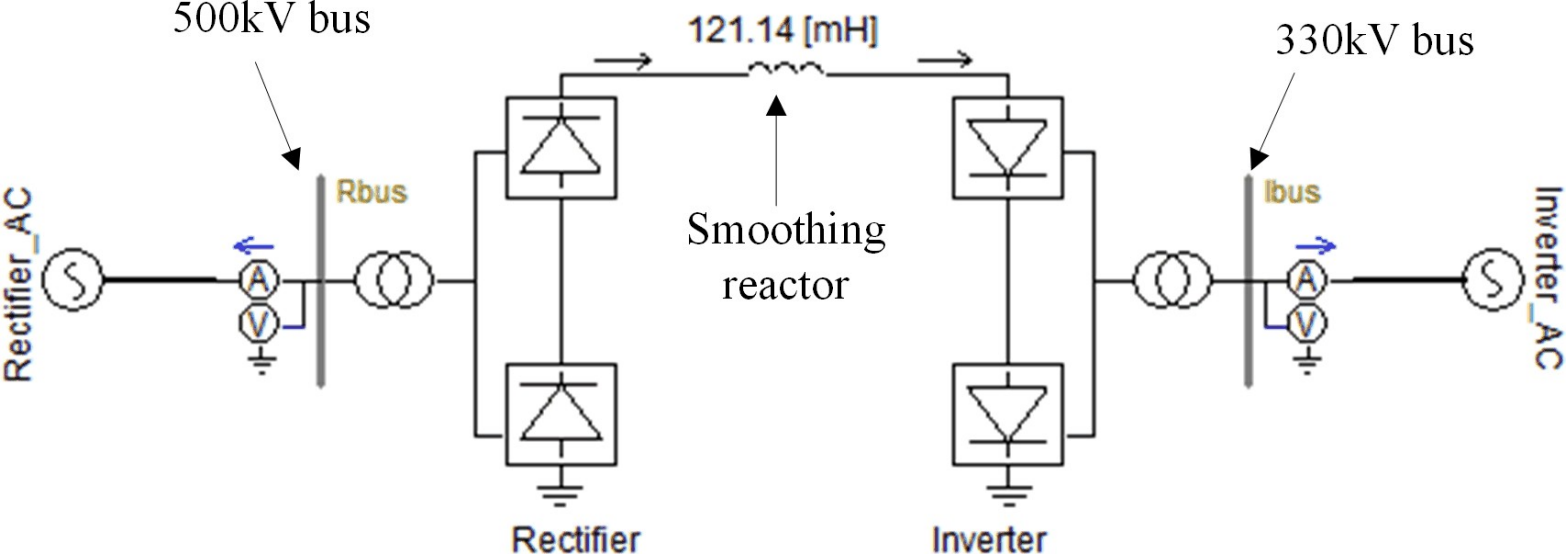
Simulation model of the back-to-back HVDC system.

A back-to-back DC power transmission system that connects a 330-kV AC system with a 500-kV AC system is examined in this paper. A double-tuned 12/24 AC filter and an AC3 high-pass filter, of which the elements rated values are listed in Table 11, are connected in parallel to the 500-kV and 330-kV AC buses of the converter station, respectively. Simulation model of this DC power transmission system has been established using PSCAD software, as shown in Fig 15.

**Table 11:** Rated element parameters of the two filters.

Filter	C1	L1	C2	L2	R1
12/24	0.9954 μF	36.374 mH	1.829 μF	19.794 mH	250 Ω
HP3	1.6046 μF	789.31 mH	12.8367 μF	—	750 Ω

**Fig 16:**
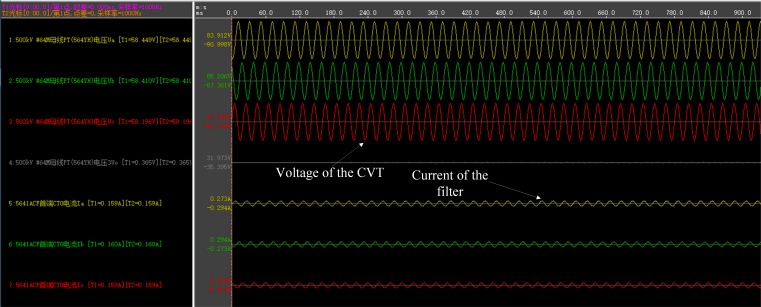
The waveforms of the filter currents and CVT voltages.

The fault recorder of the converter station has been triggered manually when the DC power transmission system is in stable operation. Three sets of operation data during several fundamental frequency cycles have been recorded, including: (1) The voltages of the buses to which the two filter banks connect, the active power of the HVDC system, the firing angle of the rectifier, and the extinction angle of the inverter. This data set is used to adjust the setting of the simulation model accurately; (2) The currents of the two filters that are used to calculate the harmonic voltages of the AC buses; (3) The voltages on the secondary side of the CVTs that are used to calculate the harmonic measurement error. Fig 16 shows the recorded waveforms of the filter currents and the CVT voltages.

**Fig 17:**
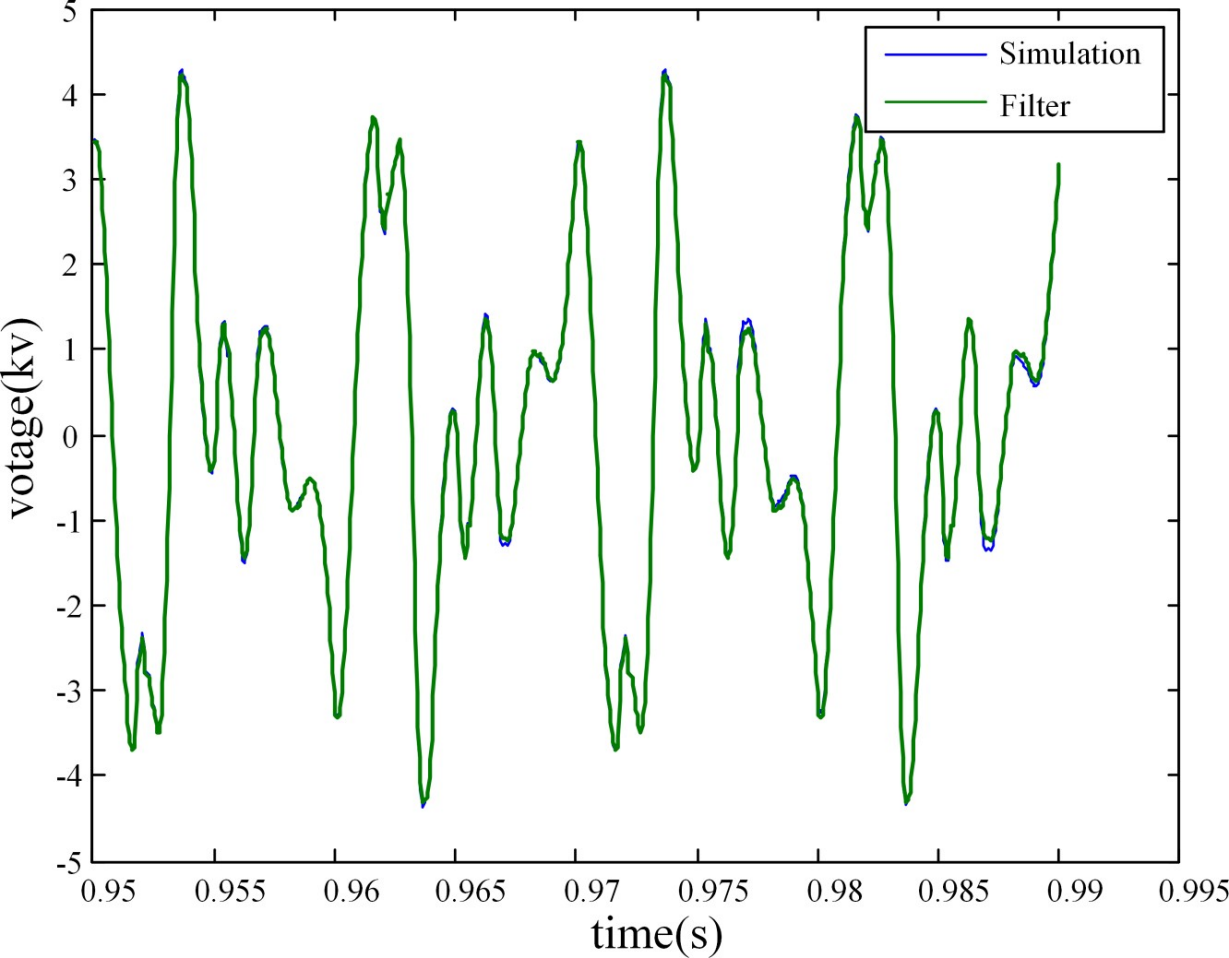
Total harmonic voltage of the 500-kV bus.

The individual harmonic voltage and the total harmonic voltage of each of the two AC buses are obtained by two ways: 1) performing simulation using the first set of data, and 2) utilizing the proposed measurement method with the second set of data. Fig 17 shows the waveform of the total harmonic voltage of the 500-kV bus, and Table 12 compares the amplitude of the harmonic voltage of each order (only those of the 2^nd^-15^th^ harmonic voltages are presented) for the two methods.

**Table 12:** Comparison of the amplitude of the individual harmonic voltage.

Harmonic order	330-kV bus			500-kV bus		
	Simulated (V)	Measured by filter(V)	Error(%)	Simulated (V)	Measured by filter(V)	Error(%)
1	275753.7	275760.5	0.02	430937.1	431032.4	0.02%
2	70.588	69.705	-1.25%	65.9753	66.8393	1.31%
3	171.463	171.892	0.25%	1511.393	1525.941	0.96%
4	2.1964	0.6385	-70.93%	0.6614	3.3531	406.93%
5	141.527	140.253	-0.90%	1914.667	1913.117	-0.08%
6	2.4024	1.6637	-30.75%	3.4162	1.601	-53.13%
7	81.4384	82.0818	0.79%	824.296	821.887	-0.29%
8	1.9653	2.0921	6.43%	2.9302	0. 9149	-68.78%
9	8.6657	8.0073	-7.60%	3.8381	1.6173	-57.86%
10	1.7756	1.5775	-11.15%	3.6735	1.9086	-48.04%
11	742.546	740.125	-0.33%	1191.311	1170.205	-1.77%
12	1.3291	0. 9902	-25.50%	1.840	0.7297	-60.32%
13	488.065	483.838	-0.87%	581.651	575.602	-1.04%
14	1.8142	1.5411	-15.09%	2.1064	1.5111	-28.28%
15	2.1306	1.9182	-9.98%	2.0933	0.6791	-67.56%

Fig 17 demonstrates that the total harmonic voltage obtained from the simulation is in very good agreement with that calculated by the filter currents. Table 12 shows that the error of the proposed method for the harmonic component with relatively large amplitude (e.g., the 3^rd^, 5^th^, 11^th^ and 13^th^ harmonic voltages) is very small, while the errors for other harmonics are relatively large due to their relatively small amplitudes. Nevertheless, the proposed method can achieve the goal of accurately measuring the harmonic voltage of a high voltage system, thus providing a convenient way for conducting the field experiments on CVTs with high voltage ratings.

### 7.3 Verification of the harmonic equivalent circuit for the 500-kV CVT

A TYD_13-0.005H_ type CVT with a rated voltage of 525/√3 kV is connected in parallel to one phase of the 500-kV AC bus of the back-to-back HVDC system. Using the method proposed in Section 2, the HEC of this CVT has been established and the parameter values of the circuit elements have been obtained. The amplitude-frequency response characteristic of the transformation ratio (*k*(*h*), *h* is the harmonic order) of this CVT is obtained through simulation and the third set of the recorded data (*U*_*cvt*2_(*h*)) presented in the previous section are used jointly to calculate the amplitude of the individual harmonic voltage of the 500-kV bus (*U*_*cvt*1_(*h*)). The proposed harmonic measurement method is employed to provide the actual values of the amplitudes of these harmonic voltages (*U*_*filter*_(*h*)) of the 500-kV bus. Table 13 lists the calculation results of the harmonic voltages with the amplitudes exceeding 100 V.

**Table 13:** Comparison of the RMS values of the individual harmonic voltages.

*h*	*U*_*filter*_(*h*) (V)	*U*_cvt2_(*h*) (V)	*k*(*h*) (dB)	*U*_cvt1_(*h*) (V)	Error (%)
1	431032.4	83.09	0.052	433618.6	0.6
3	1525.941	0.2468	-1.278	1501.236	-1.62
5	1913.117	0.3342	-0.997	1968.153	-0.88
7	821.887	0.1566	-0.133	809.478	-1.51
11	1170.205	0.3623	4.274	1164.56	-0.48
13	575.602	0.3411	10.072	561.633	0.63

In Table 13, the error of the calculated amplitude is less than 2% for each harmonic component, which demonstrates the accuracy of the obtained frequency response characteristics of the CVT. Thus, these experimental results confirm the validity of the theoretical research results concerning the harmonic equivalent circuit presented in Section 2 of this paper.

## 8. Application of the harmonic measurement error correction method

The neural network-based error correction method for harmonic measurement proposed in Section 5 is applied to the 35-kV and 525-kV CVTs with speedy saturation dampers. Table 14 lists the predicted resonant frequencies, and Table 15 compares the harmonic measurement errors before and after the correction.

**Table 14:** Prediction of the resonance frequencies.

	35kV			525kV		
	Actual value (Hz)	Predicted value (Hz)	Error (%)	Actual value (Hz)	Predicted value (Hz)	Error (%)
Resonant mode 1	2.88	2.90664	0.925	18.53	18.68751	0.85
Resonant mode 2	525.55	526.338325	0.15	713.5	714.9984	0.21
Resonant mode 3	646.96	671.285696	3.76	1582.1	1626.082	2.78

**Table 15:** Comparison of the harmonic measurement error.

h	35-kV CVT		500-kV CVT	
	Before correction	After correction	Before correction	After correction
1	0.52%	1.89%	0.92%	-1.29%
2	0.86%	2.43%	-12.71%	-2.14%
3	1.99%	-2.24%	-14.56%	-1.62%
4	4.84%	2.48%	-17.71%	-3.89%
5	8.78%	0.79%	-7.71%	2.88%
6	14.15%	-2.41%	-5.88%	0.65%
7	24.24%	-1.33%	-2.41%	-1.51%
8	37.92%	0.69%	10.01%	2.74%
9	63.55%	7.16%	14.58%	-3.42%
10	81.65%	5.54%	35.82%	-0.20%
11	-16.28%	-2.70%	63.57%	-0.48%
12	-68.30%	6.07%	117.13%	0.64%
13	-77.14%	1.45%	228.84%	0.63%
14	-68.00%	-0.13%	423.63%	-1.73%
15	-56.16%	1.43%	264.33%	-0.06%

From Table 14 it can be known that the error correction method can predict the resonant frequencies of CVTs with different voltage levels accurately, except for the relatively large error in predicting the highest resonant frequencies. Table 15 reveals that the error in the calculation of the harmonic voltage on the primary side based on the fundamental transformation ratio is very large. For example for the 525-kV CVT, the error of the amplitude of the 11^th^ harmonic voltage reaches 63.57% before correction. While after correction, the measurement errors are effectively reduced, for example the error of the 11^th^ harmonic voltage reduces to only -0.48%. These results verify that the harmonic measurement error correction method proposed in this paper is correct and effective for CVTs with various voltage levels.

## 9. Conclusions

This paper studies the mechanism for HVME and the correction method of CVTs with different types of dampers. The CVT measurement error is mainly derived from the multiple resonant modes excited by the equivalent circuit under the harmonic condition. Series resonance causes the transformation ratio amplitude at the resonance frequency to be larger than the rated value, which makes the harmonic voltage amplitude at the primary side to be smaller than the actual value, and even less than 1/5 of the actual value at most. However, the opposite is true for the parallel resonance; the calculated value under some frequencies can even reach more than dozens of times the actual value. The influence of dampers with different structures on the HVME of the CVT varies greatly. A speedy saturation damper makes little contribution to the HVME.

Through the analysis of the influence of the CVT resonant modes and different structures of dampers on the HVME, this paper presents a practical HVME error correction method based on neural network technology. The simulation results show that it can effectively fit the transformation ratio amplitude-frequency response characteristic curves of the CVT and improve the harmonic measurement accuracy of the CVT to some extent.

The experiments results verify the validity of the established harmonic equivalent circuit, the resonant frequency analysis, and the resonant frequency calculation. Relatively large error is inevitably introduced by CVTs to measure harmonic voltages. This error can be reduced to a certain extent using the harmonic measurement error correction method proposed in this paper, thus provides a basis for more better understanding the harmonic voltage characteristics of high-voltage systems.

## References

[1] Daryani N, Seyedi H. Evidence theory-based identification of aging for capacitive voltage transformers. IET Generation, Transmission & Distribution. 2016; 10(14): 3646–3653

[2] Jayachandra S, Yesuraj DJ. Modeling and simulation of capacitor voltage transformer transients using PSCAD/EMTDC. 2011 IEEE Trondheim PowerTech. 2011: 1–8.

[3] Du L, Chen B, Chen W. Research on the measurement error of capacitor voltage transformer under various insulation characteristics. 2016 IEEE International Conference on High Voltage Engineering and Application (ICHVE). 2016: 1–4.

[4] Zhu L, Ji S, Li J, Liu Y. Experimental study on the influence of the disconnecting switch operation on CVTs in UHV series compensation stations. IEEE Transaction on Dielectrics and Electrical Insulation. 2015; 22(2): 925–933.

[5] Han B, Xiang Z, Ban L, Ma Q. Study on transients and effect on capacitor voltage transformercaused by disconnector switching of UHV Series Capacitor Banks. 2014 International Conference on Power System Technology. 2014: 2293–2298.

[6] Vermeulen HJ, Dann LR, Rooijen JV. Equivalent circuit modeling of a capacitive voltage transformer for power system harmonic frequencies. IEEE Transaction on Power Delivery. 1995; 10(4): 1743–1749.

[7] Kojovic LJ, Kezunovic M, Fromen CW. A new method for the CCVT performance analysis using field measurements, signal processing and EMTP modeling. IEEE Transation on Power Delivery. 1994; 9(4): 1907–1915.

[8] Seljeseth H, Saethre EA, Ohnstad T. Voltage transformer frequency response: Measuring harmonics in Norwegian 300kV and 132kV power systems. The 8th International Conference on Harmonics and Quality of Power, ICHQP’98. 1998: 820–824.

[9] Gao H, Li Q, Yu X, Liu S. Harmonic transfer characteristic of capacitor voltage transformers. Power System Technology. 2013; 37(11): 3125–3130.

[10] Xiao Y, Fu J, Hu B, Li X, Deng C. Problems of voltage transducer in harmonic measurement. IEEE Transaction on Power Delivery. 2004; 19(3): 1483–1487.

[11] He B, Li Y, Bo ZQ. An adaptive distance relay based on transient error estimation of CVT. IEEE Transaction on Power Delivery. 2006; 21(4): 1856–1861.

[12] Ghassemi F, Gale PF, Clegg B, Cumming T, Coutts C. Method to measure CVT transfer function. IEEE Transaction on Power Delivery. 2002; 17(4): 915–920.

[13] Wu XE, Liang YS. Relationship between fractal dimensions and fractional calculus. Nonlinear Science Letters A. 2017; 8(1): 77–89.

[14] He J H. A fractional de Levie model. Computers & Mathematics with Applications. 2016 4 23 pii: 1-s2.0-S0898122116301882-main. 10.1016/j.camwa.2016.04.010

[15] Kojovic LJ, Kezunovic M, Fromen CW. A new method for the CCVT performance analysis using filed measurement, signal processing and EMTP modeling. IEEE Transaction on Power Delivery. 1994; 9(4): 1907–1914.

[16] Ghassemi F, Gale PF, Clegg B. Method to measure CVT transfer function. IEEE Transaction on Power Delivery. 2002; 17(4): 915–920.

[17] Baba Y, Ishii M. Numerical electromagnetic field analysis of unit step response characteristics of impulse voltage measuring systems. IEEE Transaction on Power Delivery. 2004; 19(1): 21–27.

[18] Ryu J, Kwon HO, Park SH, Dong WY. A square patch capacitive voltage divider for measuring high-voltage ultra wide band pluses in a coaxial pluse forming line. IEEE Transaction on Instrumentation and Measurement. 2016; 65(3): 680–684.

[19] Ghassemi F, Gale P, Cumming T, Courts C. Harmonic voltage measurements using CVT. IEEE Transaction on Power Delivery. 2005; 20(1): 443–449.

[20] Vermeulen HJ, Davel P. Voltage harmonic distortion measurements using capacitive voltage transformers. IEEE Africon. 1996; 2: 1012–1017.

[21] Miao Y, Cheng H. An optimal reactive power control strategy for UHVAC/DC hybrid system in east china grid. IEEE Transaction on Smart Grid. 2016; 7(1): 392–399.

[22] Li Y, Luo L, Rehtanz C, Yang D, Ruberg S. Harmonic transfer characteristics of a new HVDC system based on an inductive filtering method. IEEE Transaction on Power Electronics. 2012; 27(5): 2273–2283.

[23] Tziouvaras DA, McLaren P, Alexander G, Dawson D, Esztergalyos J, Formen C, et al Mathematical models for current, voltage, and coupling capacitor voltage transformers. IEEE Transaction on Power Delivery. 2000; 15(1): 62–72.

